# Efficient Cholesteric Liquid Crystal Waveguide Polarizing Splitters for High‐Sensitivity Chip‐Scale Atomic Magnetometry

**DOI:** 10.1002/advs.77320

**Published:** 2026-08-27

**Authors:** Zhibo Cui, Chuang Wang, Xiangyang Zhou, Gaopu Hou, Jinsheng Hu, Mao Ye, Xu Xiao, Zhen Chai, Yishi Weng, Yuning Zhang

**Affiliations:** ^1^ Key Laboratory of Ultra‐Weak Magnetic Field Measurement Technology Ministry of Education School of Instrumentation and Optoelectronic Engineering Beihang University Beijing China; ^2^ Institute of Large‐scale Scientific Facility and Centre for Zero Magnetic Field Science Beihang University Beijing China; ^3^ Beihang Hangzhou Innovation Institute Hangzhou China; ^4^ Hefei National Laboratory Hefei China; ^5^ Joint International Research Laboratory of Information Display and Visualization School of Electronic Science and Engineering Southeast University Nanjing China

**Keywords:** atomic magnetometers, biomagnetic imaging, cholesteric liquid crystals, quantum sensing, waveguide polarization splitters

## Abstract

High‐sensitivity spin‐exchange relaxation‐free atomic magnetometers represent the frontier of quantum sensing, yet conventional bulky polarizing beam splitters (PBSs) remain a critical barrier to chip‐scale integration. Here, we report an integrated polarimetry architecture based on a cholesteric liquid crystal (CLC) waveguide polarization splitter (WPS). By leveraging the synergy between the Bragg‐selective reflection of patterned CLCs and total‐internal‐reflection‐induced helicity inversion, this approach enables the efficient spatial decoupling and parallel emission of orthogonal circular polarizations within an ultrathin profile. We developed two application‐oriented WPS designs: a monolithic architecture optimized for high efficiency (90%) and architectural simplicity, and a cascaded architecture tailored for superior extinction ratios (141/134) and geometric symmetry. Experimental results show that the monolithic WPS reduces the optical system volume to merely 3.3% of traditional PBS‐based setups while achieving a magnetic sensitivity of 13 fT/Hz^1/2^. This sensitivity is on par with traditional bulky polarimetry schemes and surpasses existing state‐of‐the‐art integrated solutions. Furthermore, the CLC‐WPS is compatible with established liquid crystal manufacturing processes, offering a scalable and cost‐effective fabrication route obviating the need for expensive nanolithography. This work paves the way for mass‐producible, high‐performance quantum sensors, with broad implications for high‐resolution biomagnetic imaging systems.

## Graphical abstract

An integrated polarimetry architecture based on a cholesteric liquid crystal waveguide polarization splitter overcomes the volumetric bottleneck in chip‐scale atomic magnetometers. By leveraging Bragg‐selective reflection and total‐internal‐reflection‐induced helicity inversion, this ultrathin device enables efficient circular polarization splitting. It reduces optical path volume to merely 3.3% while maintaining high magnetic sensitivity (13 fT/Hz^1/2^), advancing scalable quantum sensors.

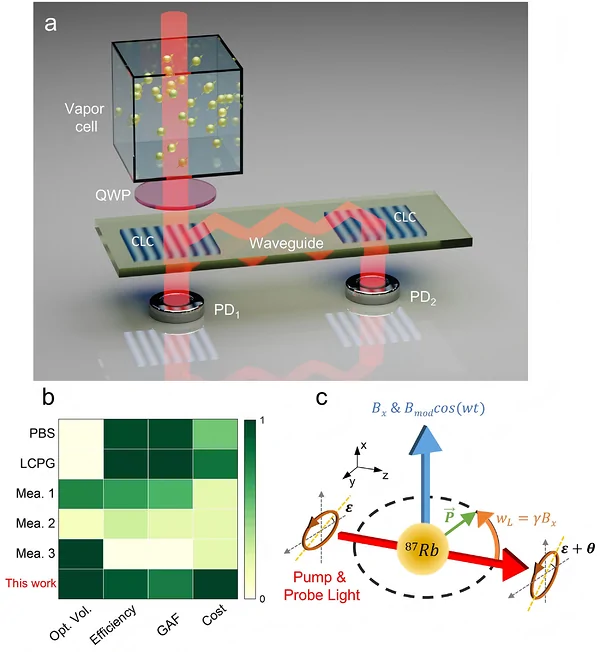

## 1 Introduction

Quantum precision measurements strive to surpass the classical shot‐noise limit and approach the fundamental Heisenberg limit [1, 2]. As a representative platform, spin‐exchange relaxation‐free (SERF) atomic magnetometers (AMs) have achieved sensitivities at the sub‐fT Hz^−1/2^ level [3, 4], catalyzing a broad spectrum of applications. These range from emerging‐material characterization [5, 6] to high‐resolution biomedical imaging [7, 8, 9], and extend from macroscopic geomagnetic anomaly monitoring [10, 11] to microscopic dark matter searches [12, 13, 14]. Spurred by the growing demand for portable instrumentation and high‐density sensor arrays, the field is rapidly evolving toward compact, fully integrated architectures that maintain high sensitivity.

Although AMs are implemented across various configurations, achieving superior performance typically relies on polarization detection, which leverages balanced differential techniques to effectively suppress common‐mode technical noise and significantly outperform optical absorption detection [15, 16]. Nevertheless, conventional polarimetric architectures rely heavily on multiple discrete bulk optics, such as polarizing beam splitters (PBSs) and reflecting prisms. This discrete configuration is inherently limited by mechanical instability, considerable device footprints, and meticulous alignment procedures. Furthermore, the reliance on macroscopic optical components is fundamentally incompatible with the scalable, cost‐effective manufacturing processes required for next‐generation sensors [17, 18]. Consequently, while the development of vertical‐cavity surface‐emitting lasers and microfabricated vapor cells has significantly accelerated miniaturization efforts [19, 20, 21], the bulky polarization detection system remains the unresolved bottleneck hindering high‐performance AM integration.

Metasurfaces, characterized by their subwavelength profiles and versatile light‐manipulation capabilities, have emerged as a promising platform for addressing these challenges [22, 23]. Existing polarimetry strategies generally fall into two distinct categories (detailed in Note S1 and Figure S1). The first utilizes free‐space architectures incorporating diffractive metasurfaces or polarization metalenses to separate polarization states via angular deflection [24, 25, 26]. However, this approach imposes an inherent constraint: substantial propagation distances are required to spatially resolve the deflected beams, thereby negating the volumetric advantage at the system level. The second category involves waveguide‐integrated approaches that strive for fully on‐chip operation [17, 27], yet they are often constrained by severe coupling and propagation losses. Crucially, magnetic sensitivity is compromised significantly as the vapor cell volume is reduced, since wall collisions of alkali atoms markedly accelerate the spin‐relaxation rate [28, 29]. Consequently, the micrometer‐scale mode profiles of integrated waveguides are intrinsically mismatched with the millimeter‐scale interaction volumes required for high‐performance AMs [30, 31]. In addition, both categories suffer from a critical fabrication bottleneck: these nanostructured devices rely almost exclusively on costly, low‐throughput nanolithography [32, 33]. Addressing this dilemma necessitates an approach that bridges high‐performance optical integration with scalable, cost‐effective manufacturing.

Recent advances demonstrate that replacing conventional bulky PBS cubes with ultracompact planar waveguiding architectures can dramatically compress the optical volume while maintaining high light throughput [34]. Liquid crystals (LCs) offer a versatile material platform for integrated photonics owing to their high optical transmittance, large birefringence, and continuously programmable Pancharatnam–Berry (PB) phase [35, 36, 37, 38]. Among these, cholesteric liquid crystals (CLCs) have emerged as a focal point in chiral photonics and integrated flat optics. Their unique periodic helical superstructure enables helicity‐dependent Bragg reflection of circularly polarized (CP) light [39], a property successfully harnessed to develop high‐performance diffractive waveguides for augmented reality (AR) displays [40, 41, 42]. Inspired by these technological strides, and recognizing the convergent requirements for extreme miniaturization, high efficiency, and low cost shared by both fields, we identify CLC‐based waveguides as a promising solution for integrated AMs. However, further tapping into the unexplored potential of CLC waveguides for high‐precision polarimetry remains essential to realize the transition from display‐grade optics to quantum‐grade sensing.

**FIGURE 1:**
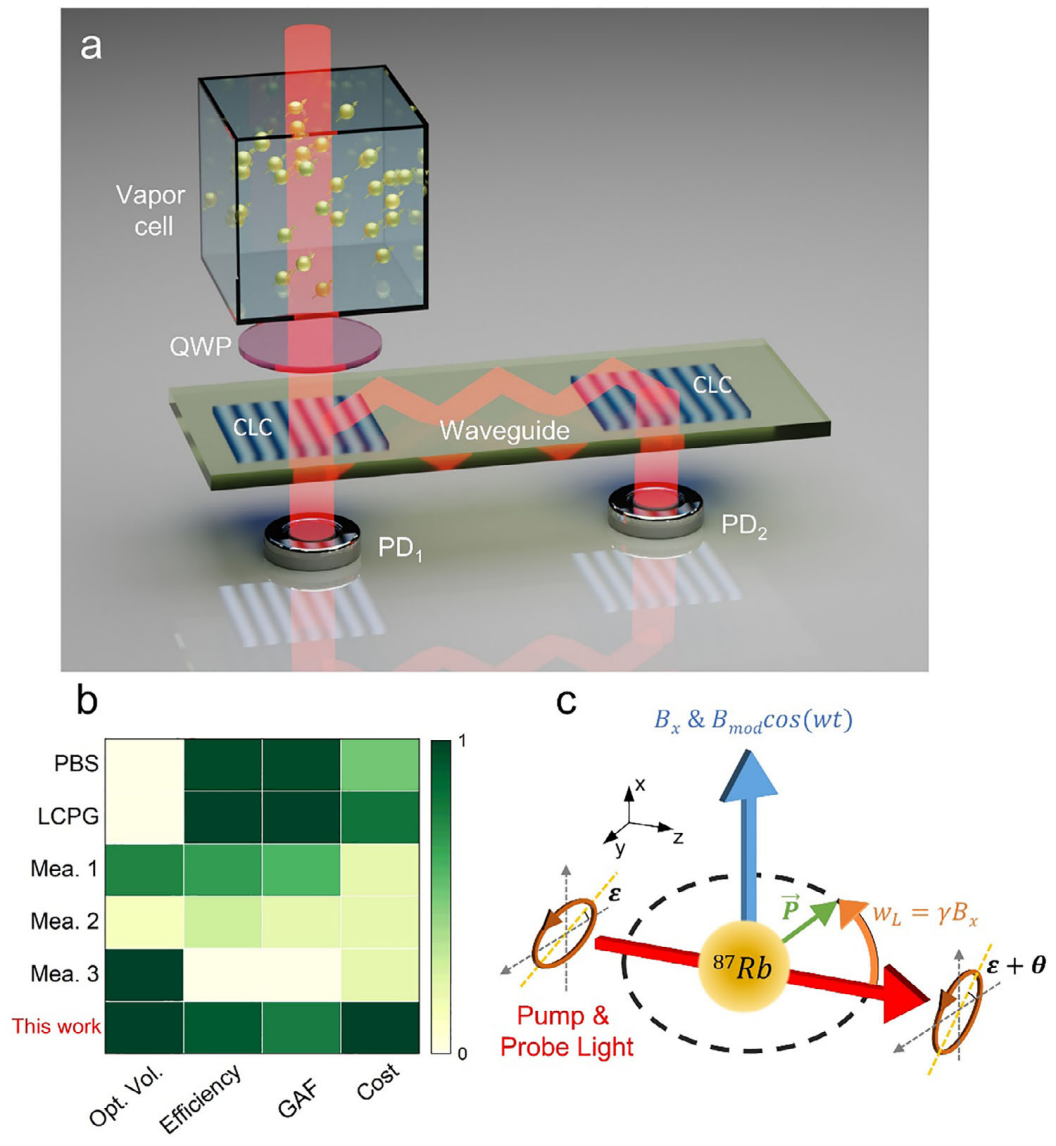
Integrated polarimetry architecture based on the cholesteric liquid crystal waveguide polarization splitter (CLC‐WPS) for atomic magnetometers (AMs). (a) Schematic illustration of the AM signal detection utilizing a CLC‐WPS. The device functions as a polarization‐selective element at the 87Rb D1 transition wavelength (795 nm), efficiently discriminating between left‐ and right‐circularly polarized (LCP/RCP) light. QWP: quarter‐wave plate; PD: photodetector. (b) Comprehensive performance benchmark comparing the proposed CLC‐WPS architecture with conventional polarizing beam splitters (PBS) and other state‐of‐the‐art integrated polarimetry devices across key metrics: optical volume, efficiency, geometric attenuation factor (GAF), and cost. To facilitate comparison, all metrics are normalized such that a larger value indicates superior performance (e.g., a higher score for volume and cost corresponds to a smaller physical footprint and lower manufacturing expense, respectively). Data for LCPG, Mea. 1, Mea. 2, and Mea. 3 are adapted from Refs. [43], [25], [24], and [27], respectively. (c) Schematic principle of the single‐beam elliptically polarized atomic magnetometer. This configuration enables simultaneous atomic optical pumping and polarimetric probing using a single light beam.

In this work, we address the aforementioned challenges by proposing an integrated polarimetry architecture for high‐sensitivity AMs based on a monolithic waveguide polarization splitter (M‐WPS), as illustrated in Figure 1a. Specifically, the optimized CLC structure acts as a bifunctional element, serving as both a polarization‐selective in‐coupler and a spatial splitter. At the ^8^ ^7^Rb D1 transition wavelength (795 nm), the device efficiently discriminates left‐ and right‐circularly polarized (LCP/RCP) components; it diffracts the LCP component into the planar waveguide while allowing the RCP component to pass through directly. Subsequently, this guided LCP light propagates via total internal reflection (TIR) and is out‐coupled vertically by the same structure, thereby enabling high‐efficiency polarization splitting and parallel emission within an ultra‐compact footprint. Figure 1b provides a comprehensive benchmark of the M‐WPS against conventional PBS setups and state‐of‐the‐art integrated splitters [24, 25, 27, 43], demonstrating its superior balance across efficiency, attenuation, and optical path volume (detailed in Table S1). Notably, our scheme reduces the detection system volume to ∼3.3% of a traditional PBS‐based setup (based on a commercial off‐the‐shelf micro‐PBS baseline with a side length of 3.2 mm), underscoring its transformative potential for AM integration.

To further enhance the extinction ratio, we developed a cascaded architecture (C‐WPS) utilizing two CLC layers with opposite helicities. This configuration significantly suppresses polarization crosstalk in the RCP channel while maintaining geometric symmetry. To validate the efficacy of our platform, we implemented single‐beam elliptically polarized atomic magnetometers (EPAMs) operating in the SERF regime using these WPS architectures. The M‐WPS and C‐WPS achieved magnetic sensitivities of 13 and 19 fT/Hz^1/2^, respectively. Remarkably, the M‐WPS performance is on par with bulky, macroscopic PBS‐based detection, while achieving a 56.7% improvement in the intrinsic geometric attenuation factor over existing metasurface‐based integrated solutions. Crucially, these devices are compatible with large‐area, low‐cost fabrication via established liquid crystal manufacturing processes, paving the way for the scalable production of chip‐scale AMs for high‐resolution biomagnetic imaging and quantum precision measurements.

## 2 Design and Methods

### 2.1 Operating Mechanism of the Integrated EPAM

The compact, single‐beam EPAM operating in the SERF regime utilizes a single elliptically polarized (EP) beam with an initial ellipticity angle  *ε* and an orientation angle ϕ, tuned to the 795 nm D1 transition of ^8^ ^7^Rb atoms and propagating along the *z*‐axis. This configuration serves a dual purpose: it simultaneously performs optical pumping and polarimetric probing, as schematically illustrated in Figure 1c. The CP component of the beam optically pumps the ^8^ ^7^Rb atoms, establishing a macroscopic spin‐polarized ensemble along the propagation axis [44]. Subjected to an external transverse magnetic field *B_x_* applied along the *x*‐axis, this spin ensemble undergoes Larmor precession around the *x*‐axis at a frequency of ω_*L*_ = γ*B_x_*, where γ = 7*Hz*/*nT* denotes the effective gyromagnetic ratio of the ^8^ ^7^Rb atomic spin.

This precession, in turn, induces a time‐varying circular dichroism and birefringence in the atomic vapor, causing the LCP and RCP components of the probe beam to experience distinct complex refractive indices, expressed as:

(1) $$\left\{\begin{array}{c} n_{r}=1+\kappa \left(1+P_{z}\right)L\left(v\right)/v\\ n_{1}=1+\kappa \left(1-P_{z}\right)L\left(v\right)/v \end{array}\right.$$

where *L*(*v*) = 1/(*v*_0_ − *v* − *i*Δ*v*/2) represents the complex Lorentzian lineshape function centered at the atomic D1 transition frequency *v*_0_, with a full width at half maximum of Δ*v*. The variable *P_z_* denotes the *z*‐component of the atomic spin polarization, which is a function of the transverse external magnetic field *B_x_*. The analytical derivation of the relationship between *P_z_* and *B_x_* is elaborated in Note S2. Furthermore, the constant is defined as κ = *nr_e_c* ^2^ *f*/4π, where *n* is the atomic number density of the alkali‐metal vapor, *r_e_* is the classical electron radius, and *f* ≈ 1/3 is the oscillator strength of the D1 transition. Subsequently, the linearly polarized component of the EP beam functions as the probe beam. Under the influence of magnetically induced circular birefringence, the polarization plane of the linearly polarized light interacts with the spin‐polarized alkali‐metal ensemble and undergoes paramagnetic Faraday rotation. The resulting rotation, referred to as the optical rotation angle θ, can be expressed as follows:

(2) $$\theta =\frac{\pi }{\lambda }\left(n_{r}-n_{l}\right)l=\frac{nlr_{e}cfRe\left[L\left(v\right)\right]}{2}P_{z}$$

where *l* is the interior length of the vapor cell.

To determine *B_x_*, a balanced differential detection scheme is utilized to measure θ, which suppresses common‐mode noise from laser fluctuations and significantly enhances detection sensitivity. Unlike traditional PBS‐based architectures relying on linear polarization bases, our configuration employs circular polarization basis decomposition. This methodology facilitates the integration of LC optics and circumvents the inherent design and manufacturing complexities associated with conventional linear polarization beam splitters. To provide a rigorous theoretical framework for system optimization, a comprehensive analytical model describing the output response of the CLC‐WPS‐based EPAM is detailed in Note S3 and Figure S2. According to this model, the differential output signal *D* of the magnetometer is expressed as:

(3) D∝ξsin4ε

where ξ is defined as the system effective attenuation factor. The EPAM output amplitude is fundamentally linked to the incident ellipticity ε, which dictates the power allocation between optical pumping and probing. This balance determines the steady‐state atomic polarization and the subsequent polarimetric response. The signal maximizes at ε = 22.5°, where beam power is equally partitioned between the two functions. Moreover, the signal strength scales with the system effective attenuation factor defined by the throughput efficiency and extinction ratio of the beam splitter channels. Analysis reveals that magnetometer performance is primarily sensitive to operating efficiency, while the extinction ratio's influence is marginal. These findings provide a key design metric for optimizing the CLC‐WPS architecture to attain peak sensitivity.

### 2.2 Design and Theoretical Characterization of the CLC‐WPSs

**FIGURE 2:**
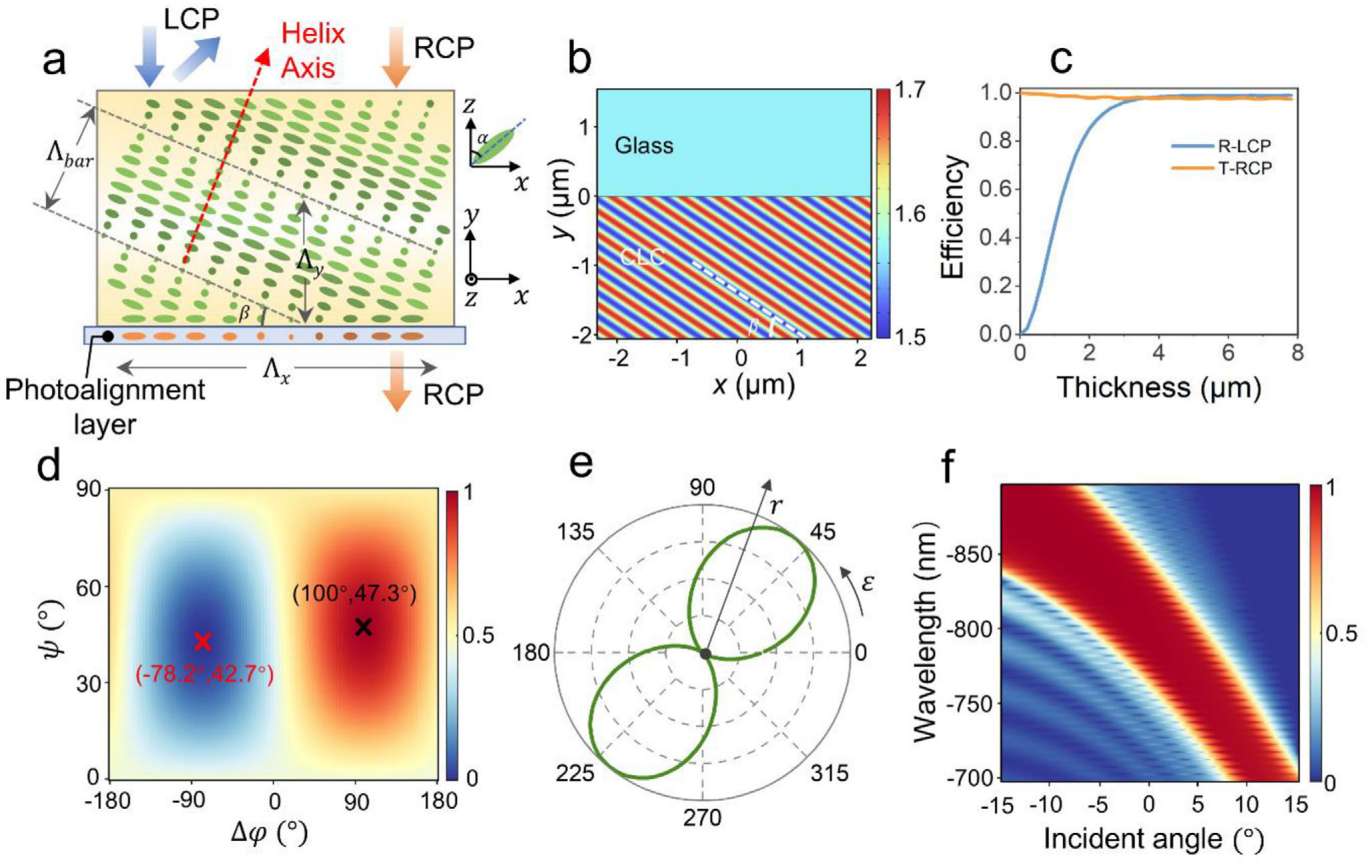
Structural model and polarization characteristics of the slanted left‐handed cholesteric liquid crystal (S‐CLC). (a) Schematic illustration of the S‐CLC molecular structure model, where the liquid crystal director naturally relaxes into a slanted helical configuration. (b) Simulated refractive index distribution of the structure in the *xy*‐plane, revealing a series of slanted iso‐refractive‐index planes with a specific tilt angle β. (c) Simulated diffraction efficiency under LCP incidence and transmittance under RCP incidence as a function of the S‐CLC layer thickness. (d) Spatial distribution of diffraction efficiency under varying incident polarization states (parameterized by the Jones vector), where Δφ and ψ denote the phase difference and the amplitude auxiliary angle, respectively. (e) Diffraction efficiency curve as a function of the ellipticity angle ε, exhibiting a peak response at ε = 50.5° due to the intrinsic symmetry breaking of the slanted structure. (f) Simulated angular and spectral response of the device under LCP illumination.

The self‐assembled, nanoscale helical structure of CLCs endows the medium with a unique polarization‐selective response [45]. Specifically, the CLC structure exhibits high‐efficiency Bragg reflection for CP light sharing the same handedness as the helix while remaining highly transparent to the orthogonal state. To distinguish between different chiralities, right‐handed and left‐handed CLCs are hereafter denoted as R‐CLCs and S‐CLCs, respectively. The patterned CLCs employed here are characterized by a dual periodicity comprising a horizontal period Λ_*x*_ and a vertical period Λ_*y*_. While Λ_*x*_ is strictly imposed by the pre‐patterned alignment layer, Λ_*y*_ is precisely tunable via the chiral dopant concentration. Although earlier models assumed a simplified planar distribution, recent studies indicate that the LC director spontaneously relaxes into a slanted helical configuration to minimize the system's elastic free energy [46], as schematically illustrated in Figure 2a.

To quantitatively describe this microstructure, the spatial distribution of the LC director's azimuthal angle α(*x*, *y*), defined as the angle between the director and the *z*‐axis, is expressed as:

(4) $$\alpha \left(x,y\right)=\frac{\pi x}{\Lambda_{x}}+\frac{\pi y}{\Lambda_{y}}$$

Consequently, the LC director exhibits a 3D periodic rotation, inducing a periodic modulation of the dielectric tensor. Figure 2b depicts the resulting refractive index distribution, revealing that the continuous rotation of the director field is optically analogous to a series of slanted iso‐index planes. These planes exhibit a tilt angle $\beta =\arctan(\Lambda_{y}/\Lambda_{x})$ relative to the substrate. Structurally, this arrangement constitutes a slanted Bragg grating and is thus also referred to as a polarization volume grating [47].

The optical behavior of the device is governed by Bragg diffraction. When the probe beam is incident at an angle θ_*in*_ relative to the surface normal, the CLC selectively reflects the CP component sharing the same handedness as its own helix. This diffraction process adheres to the generalized Bragg condition:

(5) $$2n_{\mathrm{eff}}\Lambda_{bra}\cos\left(\beta -\theta_{in}\right)=\lambda_{bra}$$

where λ_bra_ represents the target wavelength and $n_{eff}=\sqrt{({n_{e}}^{2}+2{n_{o}}^{2})/3}$ denotes the average effective refractive index of the LC medium. Furthermore, based on the specular‐like reflection geometry originating from the tilted planes, the resulting diffraction angle is determined by θ_*d*if_ = θ_*in*_ + 2β. This fundamental relationship indicates that by precisely tuning the tilt angle β, the device can effectively couple the incident light into the waveguide at a diffraction angle that exceeds the critical angle for TIR.

To realize the design, the reactive mesogen RM257 (*n_o_* = 1.508, *n_e_* = 1.687 at 795 nm) was employed, resulting in a volume‐averaged effective refractive index of *n*_eff_ ≈ 1.5667. An index‐matched glass substrate was selected as the waveguide to minimize interfacial Fresnel reflections and coupling losses. To satisfy the TIR condition, the target diffraction angle was set to θ_*d*i*f*_ = 56°, requiring a grating tilt angle of β = 28° for a normally incident probe beam. Based on the generalized Bragg condition, the effective period Λ_*bra*_ is determined to be 287 nm, which dictates the horizontal and vertical periods as Λ_*x*_ = 612 nm and Λ_*y*_ = 325 nm respectively.

To numerically investigate the optical performance of the patterned CLC, a 2D finite‐element method model of the S‐CLC was established using COMSOL Multiphysics. Figure 2c illustrates the simulated diffraction efficiency for incident LCP and the transmittance for incident RCP as a function of the CLC layer thickness. The LCP diffraction efficiency increases monotonically with thickness and exceeds 0.98 at 4 µm, demonstrating that sufficient thickness is essential to reach the strong‐Bragg regime. In contrast, the RCP component exhibits near‐unity transmission independent of the layer thickness. Consequently, a CLC film thickness of 4.8 *µm* was selected to ensure optimal device performance and high diffraction efficiency.

In conventional planar CLC models, the eigen‐polarizations are strictly circular. However, in the slanted configuration explored here, the tilted helical axis induces a coordinate transformation of the dielectric tensor (detailed in Note S4), thereby modulating the polarization response. To quantitatively analyze this effect, we utilized rigorous coupled‐wave analysis to map the diffraction efficiency across the entire polarization space. An arbitrary input polarization state is parameterized via the Jones vector *E_in_* = [*cos*ψ, *sin*ψ*e* ^*i*Δφ^]^*T*^, where ψ ∈ [0°, 90°] denotes the amplitude auxiliary angle, and Δφ ∈ [− 180°, 180°] represents the phase difference between the orthogonal components. Figure 2d illustrates the spatial distribution of diffraction efficiency under varying incident polarization states in the slanted model. The results reveal that the optimal polarization states deviate from perfect circularity. Specifically, the peak efficiency is attained at (Δφ,      ψ) = (100°, 47.3°) rather than the LCP state (90°, 45°), while the minimum occurs at (− 78.2°, 42.7°) instead of the RCP state (− 90°, 45°). To visualize this deviation, we utilize the ellipticity angle ε defined in Section 2.1, which is analytically related to the Jones parameters by sin (2ε) = *sin* (2ψ)*sin* (Δφ). Figure 2e plots the diffraction efficiency as a function of ε. The results show that the peak diffraction efficiency occurs at ε ≈ 50.5°, deviating by approximately 5.5° from the LCP state (45°). Similarly, the minimum efficiency corresponds to ε ≈ 128.7°, deviating by approximately 6.3° from the RCP state (135°). These shifts provide physical evidence of the symmetry breaking induced by the slanted grating vector.

Furthermore, we simulated the angular and spectral response under LCP illumination, as shown in Figure 2f. The results demonstrate a robust operational tolerance, with the diffraction efficiency exceeding 95% across a 60 nm spectral bandwidth and a 16.7° angular bandwidth. This intrinsic stability is critical for the subsequent AM detection system, as it ensures robustness against laser frequency drift and mechanical alignment errors. Notably, under pure LCP incidence, the peak diffraction efficiency corresponds to an incidence angle of 1.8° at a wavelength of 784 nm. However, when the incident light conforms to the optimized elliptical polarization state (Δφ,      ψ) = (100°, 47.3°), the peak efficiency is perfectly restored to the design target of 795 nm and 0° incidence. These findings reveal a fundamental design principle: precisely tailoring the incident polarization to match the grating's structural symmetry is essential to maximize the performance of the waveguide architecture. Detailed scanning data are provided in Note S5 (Figure S3).

**FIGURE 3:**
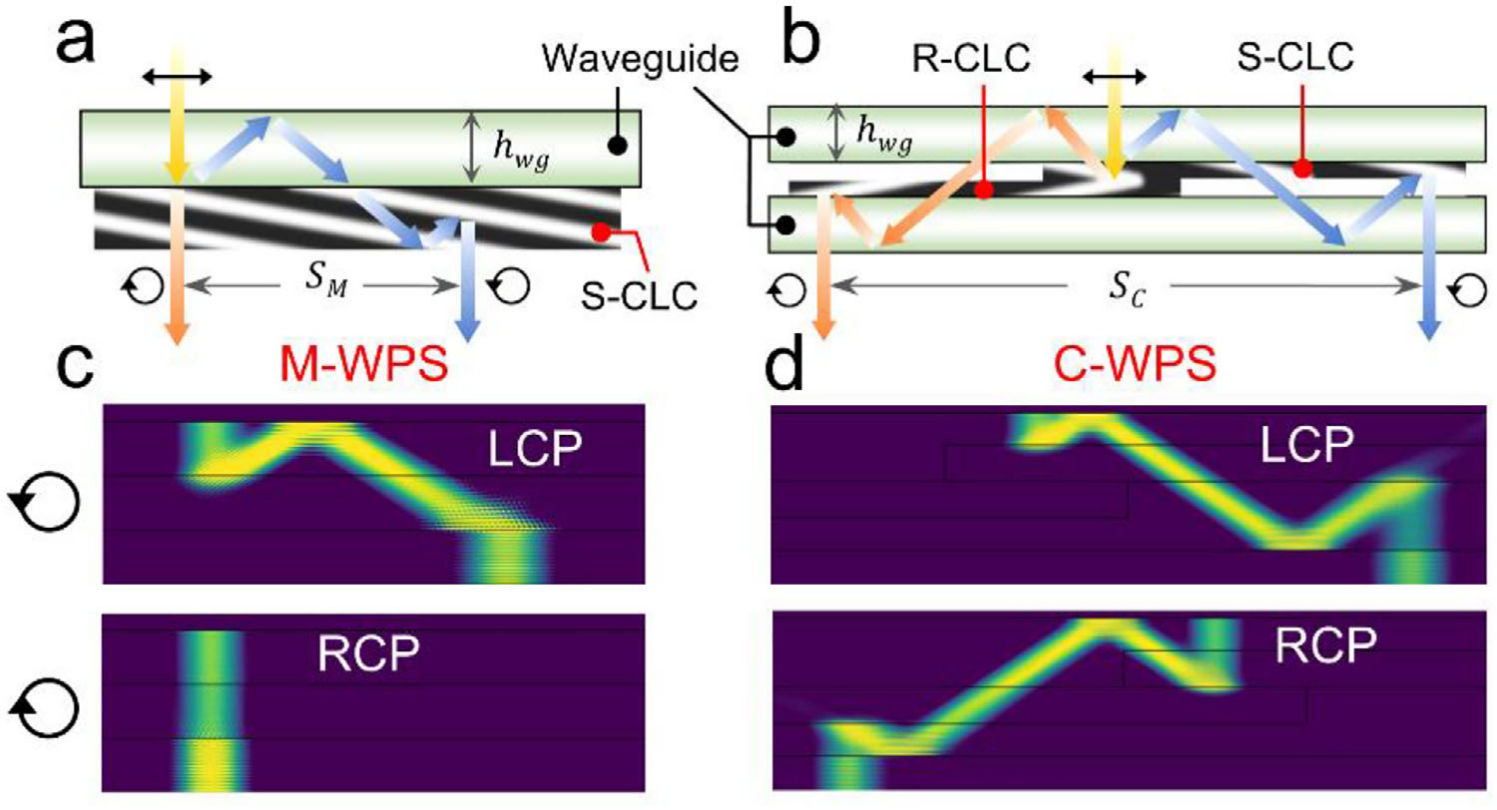
Architectures and beam‐splitting mechanisms of the M‐WPS and C‐WPS. (a) Schematic of the monolithic waveguide polarization splitter (M‐WPS) configuration, integrating a single waveguide with a bottom S‐CLC film. (b) Schematic of the cascaded waveguide polarization splitter (C‐WPS) configuration, employing a cascaded design with stacked S‐CLC and Right‐handed CLC (R‐CLC) films. This architecture strictly suppresses polarization crosstalk via a dual‐selective filtering mechanism. (c,d) Simulated light propagation field distributions for the M‐WPS and C‐WPS architectures under LCP and RCP excitation.

By exploiting the unique polarization‐selective properties of patterned CLCs in conjunction with waveguide‐mediated transport, we developed two WPS architectures for high‐precision polarization detection. Figure 3a illustrates the M‐WPS configuration, which consists of a single waveguide integrated with a bottom S‐CLC film that serves as both the in‐coupler and out‐coupler. Under normal incidence, the RCP component undergoes direct transmission due to the CLC's polarization selectivity, whereas the LCP component is diffracted at θ_*d*i*f*_ = 56° and coupled into the waveguide. Because this angle exceeds the critical angle for TIR (θ_*cri*_ ≈ 39.6°) at the glass‐air interface, the LCP light is effectively confined. The key extraction mechanism is TIR‐induced handedness inversion: the initial TIR event introduces a π phase shift, converting the LCP state into RCP. Consequently, during the second interaction with the S‐CLC layer, the reflected RCP beam propagates through without further diffraction. A subsequent TIR at the bottom CLC‐air boundary restores the polarization back to LCP. At this stage, the beam satisfies the Bragg condition at a conjugate incidence angle (− θ_*d*i*f*_), resulting in diffraction along the surface normal (0°) and vertical out‐coupling. Ultimately, the M‐WPS achieves spatial separation and parallel emission of orthogonal circular polarizations, with a separation distance of *S_M_* = 2*h_wg_* tan (θ_*d*i*f*_), where *h_wg_* denotes the waveguide thickness. The lateral beam shift within the CLC layer is neglected as the film thickness is significantly smaller than that of the waveguide.

In practice, factors such as non‐ideal LC alignment may prevent the LCP component from being fully diffracted during the initial interaction, leading to polarization crosstalk where residual LCP contaminates the transmitted RCP beam. To suppress this crosstalk and enhance the extinction ratio, we developed a C‐WPS comprising two stacked films of opposing handedness (S‐CLC and R‐CLC), as illustrated in Figure 3b, similar to multi‐twist and multi‐layer liquid crystal architectures designed for precise spectral filtering and polarization control [48]. In this architecture, the S‐CLC diffracts the LCP component into a rightward mode, while the subsequent R‐CLC diffracts the RCP component into a leftward mode. Furthermore, the inclusion of a bottom waveguide modifies the light propagation path, as the second TIR event required to restore the polarization state occurs at the lower glass‐air interface. Consequently, both guided CP beams undergo two complete TIR cycles before exiting perpendicularly, resulting in an increased separation distance of *Sc* = 8*h_wg_* tan  (θ_*dif*_).

Finally, we simulated light propagation under LCP and RCP illumination for both architectures. The resulting cross‐sectional electric‐field distributions are presented in Figure 3c,d. To clearly visualize the optical paths while reducing computational load, the structural dimensions were scaled, with the CLC layer proportion intentionally accentuated. The simulation results unambiguously confirm that both designs efficiently decouple LCP and RCP components into distinct trajectories, thereby providing compelling numerical corroboration for the proposed beam‐splitting mechanisms.

## 3 Results and Discussion

### 3.1 Fabrication of the CLC‐WPSs

**FIGURE 4:**
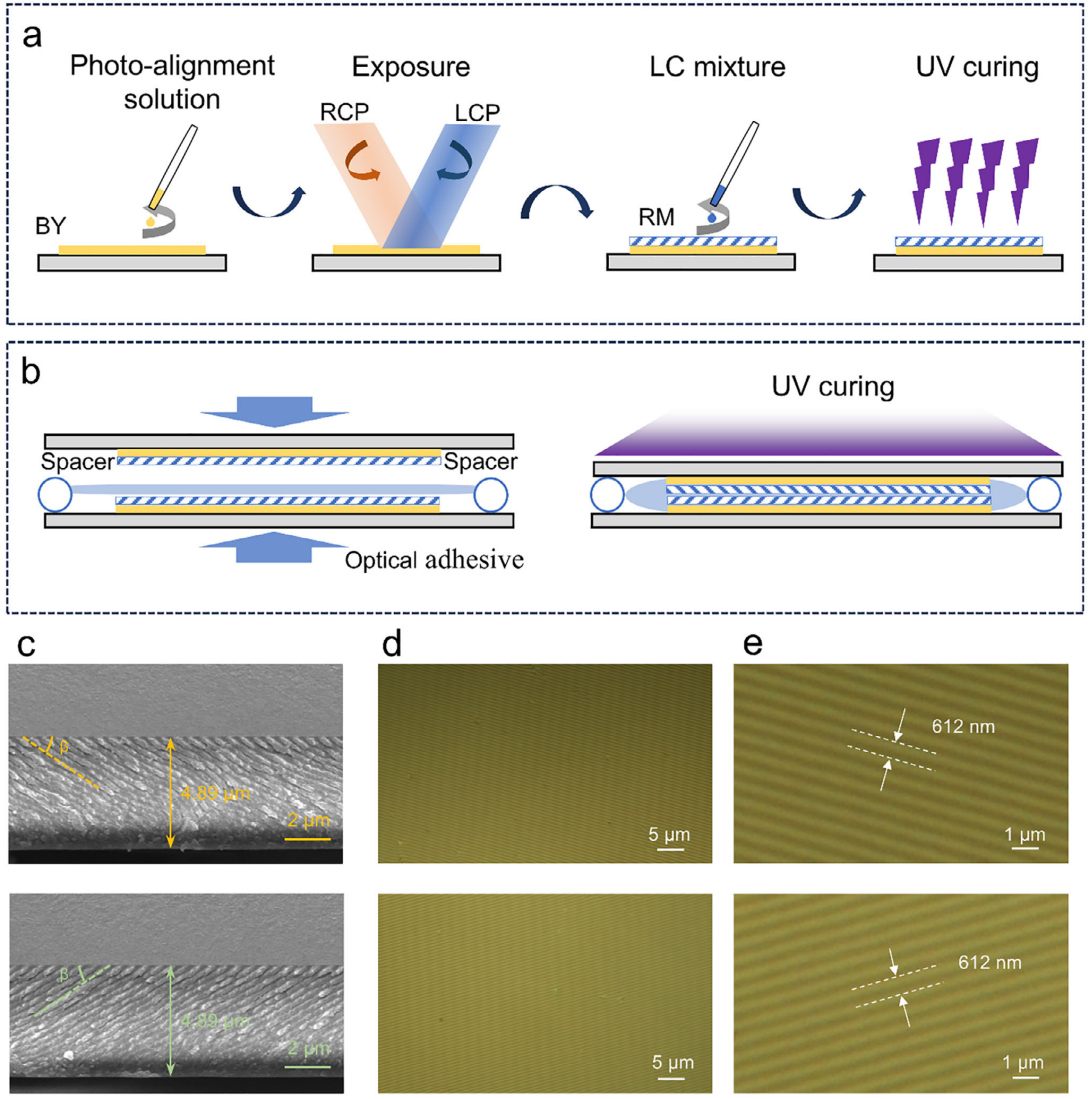
Fabrication workflow and structural characterization. (a) Schematic workflow for fabricating the patterned CLC film. BY: brilliant yellow; UV: ultraviolet. (b) Schematic of the precision lamination process employed to realize the C‐WPS architecture. (c) Cross‐sectional scanning electron microscopy image of the S‐CLC film, clearly revealing the periodic microstructure with a tilt angle of 28° and a film thickness of approximately 4.89 µm. For brevity, only the S‐CLC variant is characterized here, as the R‐CLC structure is its mirror‐symmetric counterpart. (d) Top‐down polarized optical microscopy (POM) image confirming high‐quality, large‐area, defect‐free monodomain alignment. (e) Magnified POM view showing the lateral grating period precisely measured to be 612 nm.

The fabrication workflow for the M‐WPS is schematically illustrated in Figure 4a. The process begins with the deposition of a photoalignment layer on an index‐matched glass substrate to suppress interfacial reflections. Subsequently, a periodic anchoring pattern is defined on this layer via polarization interference exposure, utilizing two intersecting LCP and RCP laser beams of equal intensity. Detailed specifications of the exposure setup are provided in Note S6 (Figure S4). Following this, the CLC precursor mixture is spin‐coated onto the patterned substrate. Driven by the synergy between the surface anchoring template and the intrinsic helical twisting power of the chiral dopant, the LC director field self‐assembles into a thermodynamically stable, 3D slanted helical configuration. Finally, UV‐induced photopolymerization solidifies the structure into a robust polymer film. Detailed fabrication protocols and material compositions are provided in the Methods section.

To construct the C‐WPS, a precision lamination process is employed, as depicted in Figure 4b. Two glass substrates coated with S‐CLC and R‐CLC films are assembled in a face‐to‐face configuration. The inter‐layer gap is filled with an optical adhesive integrated with 30 µm diameter silica spacers to ensure uniform and precise thickness control. Subsequent UV irradiation cures the adhesive, effectively encapsulating the CLC films while providing enhanced structural integrity and environmental shielding for the functional layers.

Following fabrication, the structural fidelity of the WPS samples was characterized via scanning electron microscopy (SEM) and polarized optical microscopy (POM). Figure 4c presents the cross‐sectional SEM image of the S‐CLC film. For brevity, only the S‐CLC variant is characterized here, as the R‐CLC structure is its mirror‐symmetric counterpart. This image clearly reveals a well‐defined internal periodic modulation, with a measured film thickness of 4.89 µm and a grating fringe tilt angle of 28^°^, both of which are in excellent agreement with the design parameters. Furthermore, top‐down POM imaging confirms a high‐quality, large‐area monodomain alignment with negligible defect density (Figure 4d). From the magnified view (Figure 4e), the horizontal period was determined to be 612 nm, perfectly matching the design target.

### 3.2 Optical Characterization of the CLC‐WPSs

**FIGURE 5:**
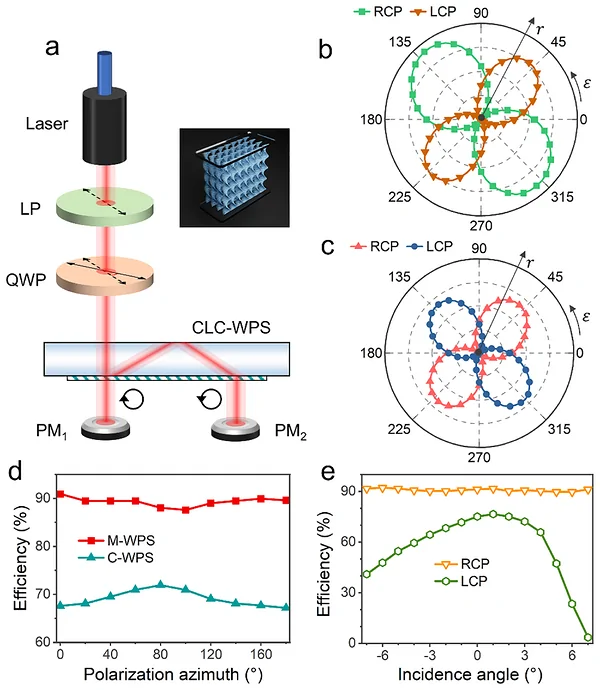
Optical performance characterization of the fabricated devices. (a) Schematic of the custom‐built optical setup used to quantify the polarization‐selective performance. LP: linear polarizer; QWP: quarter‐wave plate; PM: power meter. (b,c) Measured normalized transmission efficiencies of the decoupled LCP and RCP channels for the M‐WPS and C‐WPS, respectively, as a function of the input ellipticity angle ε. The curves exhibit characteristic sin ^2^(2ε) and cos ^2^(2ε) dependence, in excellent agreement with theoretical predictions. (d) Total optical efficiency of the M‐WPS and C‐WPS architectures as a function of the incident linear polarization orientation. (e) Angular robustness of the M‐WPS, showing the measured efficiency of the LCP and RCP channels as a function of the incident angle.

To systematically quantify the polarization‐ selective performance of the proposed architectures, a custom‐built optical characterization setup was established. As schematically illustrated in Figure 5a, a collimated 795 nm Gaussian beam was modulated by a linear polarizer (LP, LBTEK FLP25‐NIR‐M) and a quarter‐wave plate (QWP, LBTEK QWP25‐795A‐M) to produce incident light with a continuously tunable ellipticity angle ε. The output powers from the decoupled LCP and RCP channels were monitored simultaneously using two power meters (Thorlabs PM120VA) to ensure synchronized data acquisition. Figure 5b,c present the normalized transmission efficiency as a function of the angle ε for the M‐WPS and C‐WPS architectures, respectively. For both devices, the efficiencies of the LCP and RCP channels exhibit characteristic sin ^2^(2ε) and cos ^2^(2ε) dependencies, demonstrating excellent agreement with theoretical predictions. Furthermore, to quantify the polarization purity, the circular polarization extinction ratio (CPER) for each channel was defined using the intensity ratios *I* ^max ^_*RCP*_/*I* ^min^_*RCP*_ and ${I^{\max}}_{LCP}/{I^{\min}}_{LCP}$, where ${I^{\max}}_{RCP}$ represent the maximum intensities recorded for the RCP channel, and so forth. The M‐WPS yielded CPER values of 13.5/82 for the RCP/LCP channels, while the C‐WPS achieved a significantly enhanced performance of 141/134, confirming its superior capability in suppressing polarization crosstalk. To verify device‐to‐device repeatability, five independent samples were fabricated and tested under identical protocols, exhibiting high batch‐to‐batch consistency (detailed statistical data provided in Note S7 and Table S3).

Further inspection reveals a pronounced asymmetry between the LCP and RCP channels in the M‐WPS: the RCP channel exhibits a higher peak efficiency but a lower CPER relative to the LCP channel. This disparity is primarily attributed to minor imperfections in CLC self‐assembly, which lead to incomplete diffraction of the LCP component and subsequent residual leakage into the RCP channel. Notably, the C‐WPS effectively overcomes this limitation. By subjecting both channels to stringent polarization‐selective filtering through dual CLC layers, the CPER is significantly bolstered. Furthermore, the geometrically symmetric optical paths in this configuration ensure balanced propagation losses, yielding the exceptional splitting symmetry observed in Figure 5c. For both architectures, the peak efficiency of the LCP channel consistently occurs at an ellipticity angle of ε = 50°, deviating from the ideal circular state (45°). This experimental finding perfectly corroborates our simulation results in Section 2.2, validating that this polarization response shift is intrinsically induced by the symmetry breaking inherent to the slanted helical CLC architecture.

Figure 5d presents the total efficiency of both architectures as a function of the incident linear polarization orientation. The average efficiency decreases from approximately 90% for the M‐WPS to 70% for the C‐WPS. To transparently elucidate this throughput discrepancy, a quantitative optical loss budget along their respective waveguiding trajectories was established (detailed in Note S8 and Table S4). While the M‐WPS minimizes the optical path structure to achieve a high net throughput, the C‐WPS undergoes two complete TIR cycles and traverses the intermediate bonding layer six times, giving rise to cumulative absorption and interface reflection losses. Consequently, the reduced efficiency of the C‐WPS represents a necessary engineering trade‐off to realize the enhanced extinction ratio and splitting symmetry demonstrated above.

In practical applications, as alignment errors often lead to deviations from normal incidence, evaluating the angular robustness of the device is a critical performance metric. To assess this, the representative M‐WPS was mounted on a calibrated rotation stage (GTLIN R59‐360PD) for precise control of the incident angle. The incident polarization was first modulated via a QWP to maximize the transmission efficiency of the RCP and LCP channels, respectively. Subsequently, the efficiency of both channels was measured as a function of the incident angle, as plotted in Figure 5e. The results show that the RCP channel exhibits a relatively flat response, with efficiency remaining largely insensitive to the incident angle and consistently exceeding 90%. While the LCP channel is more sensitive to the incident angle, it still maintains an efficiency of over 65% across a substantial range from −4° to +3°. These findings demonstrate excellent angular tolerance, ensuring the reliability of the device for practical deployment.

To evaluate the practical operational bandwidth of the CLC‐WPS, the diffraction spectrum of the fabricated M‐WPS was experimentally characterized (detailed in Note S9 and Figure S5). Benefiting from the large LC birefringence, the measured diffraction spectrum exhibits a broad bandwidth of ∼65 nm centered around 795 nm. This broad spectral tolerance ensures that the device performance remains completely unaffected by laser frequency detuning. Furthermore, thermal cycling tests between +80°C and −10°C over 48 h revealed a negligible efficiency variation (changing from 89.2% to 88.8%), confirming that photopolymerization permanently locks the helical pitch in a thermally stable polymer matrix and ensures robust long‐term operational stability.

**FIGURE 6:**
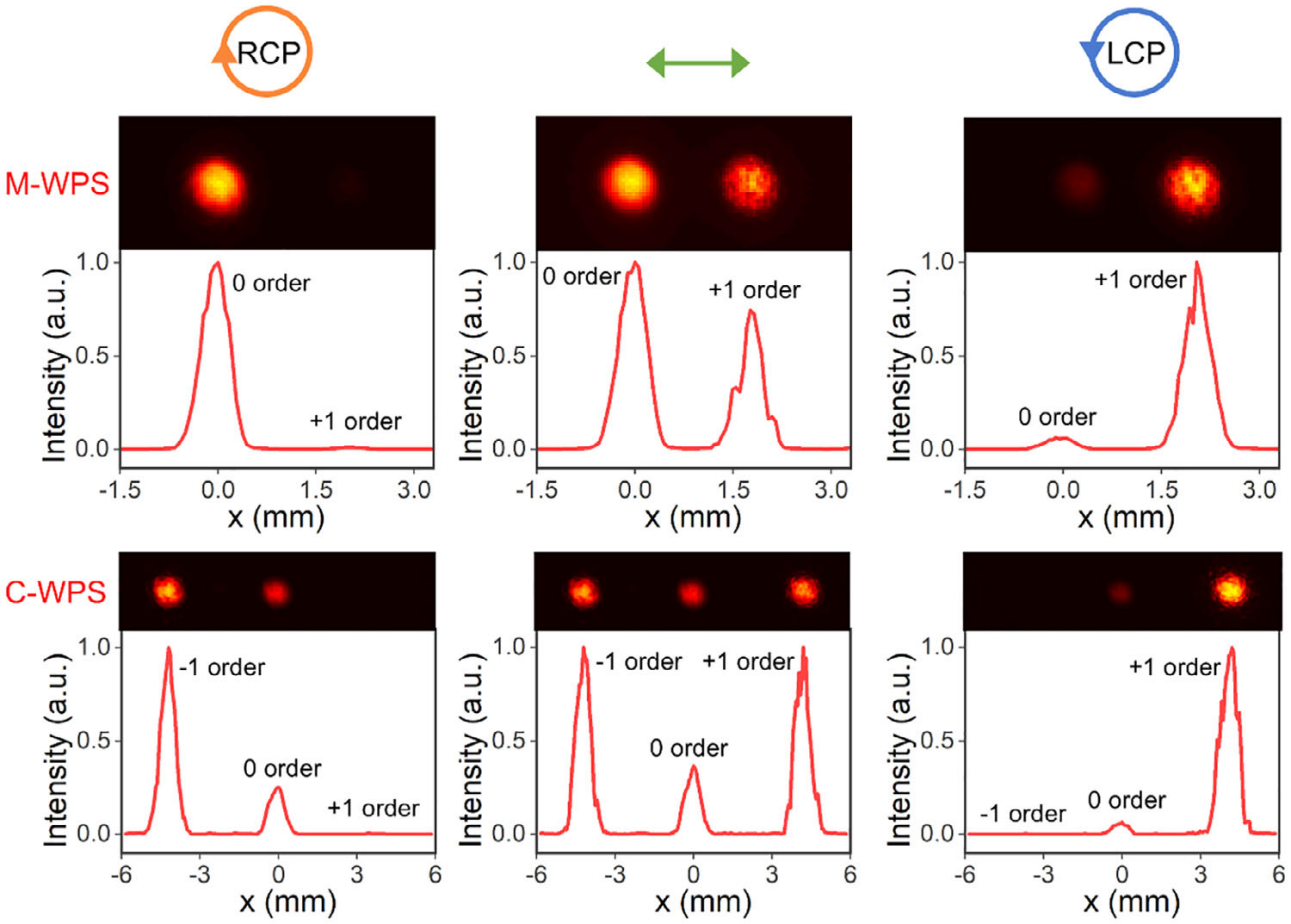
Output beam spatial profile and separation distance characterization. Transverse spatial intensity profiles of the M‐WPS and C‐WPS recorded at a propagation distance of ∼1 cm under LCP, RCP, and linear polarization states, demonstrating exceptional polarization selectivity. Superimposed normalized intensity cross‐sections along the lateral *x*‐axis confirm that both architectures maintain Gaussian‐like modes. Furthermore, the measured beam separation distances of 1.8 and 8.2 mm show excellent agreement with the theoretical design targets of 2.08 and 8.3 mm, respectively.

Finally, we characterized the transverse spatial profiles of the output beams for both the M‐WPS and C‐WPS architectures. Figure 6 displays the beam profiles recorded at a propagation distance of ∼1 cm under varying input polarization states, superimposed with their normalized intensity cross‐sections along the lateral *x*‐axis. The results confirm that the output beams in both configurations exhibit Gaussian‐like distributions, with measured separation distances of 1.8 and 8.2 mm, which are in good agreement with the theoretical design targets of 2.08 and 8.3 mm, respectively. Minor discrepancies are primarily attributed to slight alignment deviations within the experimental setup. Furthermore, a significant advantage of this architecture is the tunability of the beam separation, which can be realized via two mechanisms: first, the lateral displacement per TIR cycle can be continuously modulated by adjusting the waveguide thickness *h_wg_* and the diffraction angle θ_*dif*_; second, the total separation can be discretely defined by the spatial arrangement of the out‐couplers. This geometric flexibility allows for the customization of beam spacing to match specific detector array configurations, ensuring versatile integration into various optical sensing systems.

### 3.3 Performance of the CLC‐WPS‐Based EPAM

**FIGURE 7:**
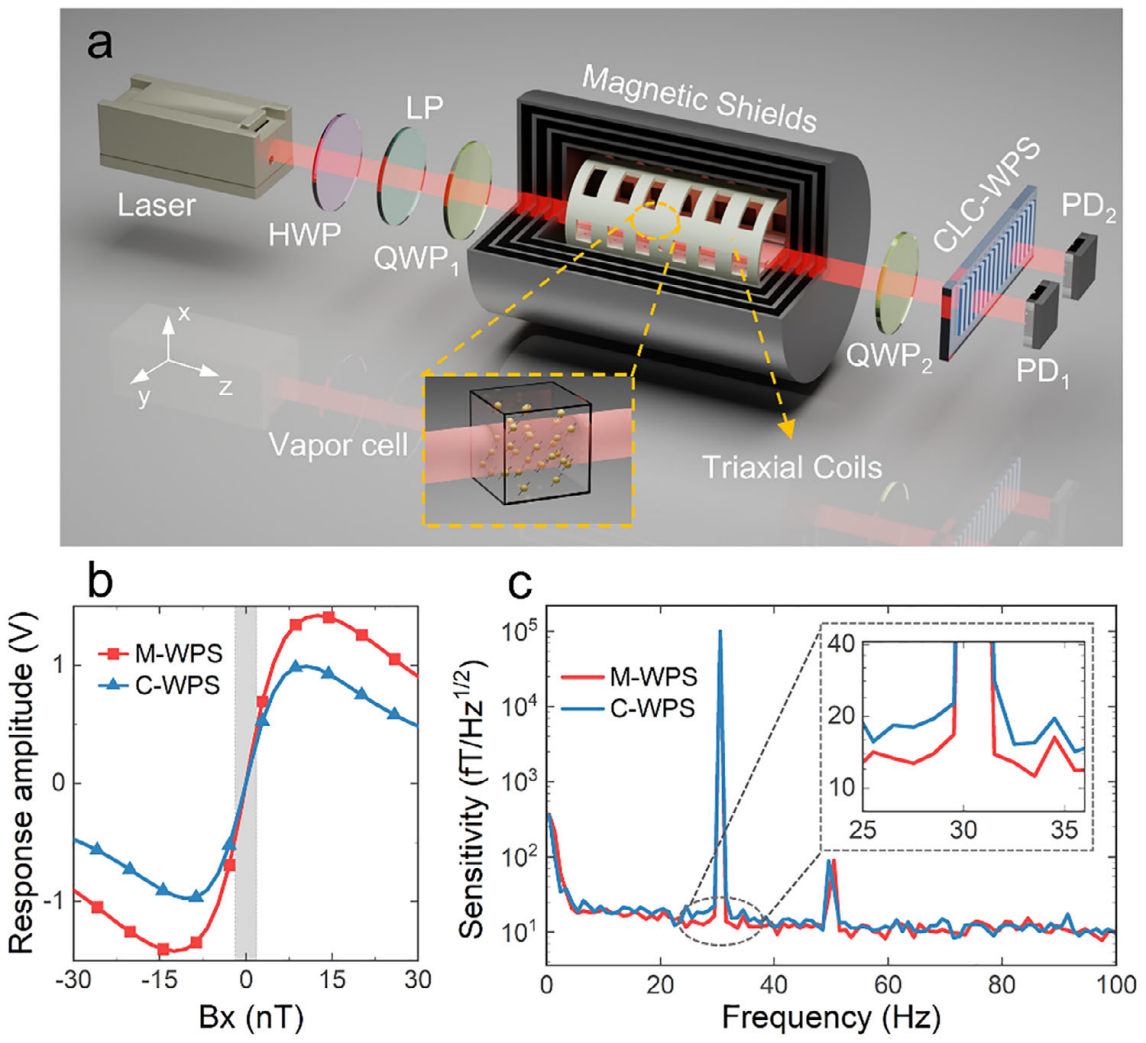
Performance characterization of the single‐beam elliptically polarized atomic magnetometer (EPAM) integrated with CLC‐WPS. (a) Schematic of the experimental setup for the CLC‐WPS‐based single‐beam EPAM. (b) Measured dispersion curves of the magnetometers integrated with M‐WPS and C‐WPS, exhibiting characteristic dispersive Lorentzian lineshapes. (c) Noise spectral density profiles for the magnetometers integrated with M‐WPS and C‐WPS, achieving magnetic sensitivities of 13 and 19 fT/Hz^1/2^ at 30.5 Hz, respectively.

To validate the practical utility of the proposed integrated polarization detection scheme, we implemented a single‐beam EPAM incorporating a CLC‐WPS, as schematically illustrated in Figure 7a. A 795 nm continuous‐wave beam from a distributed feedback (DFB) diode laser is collimated and directed through an LP and a QWP to generate an optimized EP state with an ellipticity angle of 22.5°. Upon entering a 1 cm^3^ vapor cell enriched with ^87^Rb, this EP beam executes a dual‐functional role: its circular component optically pumps the ^87^Rb atoms into a spin‐polarized ensemble, while its linear component simultaneously probes the spin precession via the magneto‐optical rotation effect. The emerging beam traverses a second QWP, translating the optical rotation into an intensity‐modulated differential signal between orthogonal circular polarizations. Here, the CLC‐WPS serves as the core polarization‐splitting element, efficiently decoupling these orthogonal components and guiding them onto a balanced detection module consisting of two photodiodes (PDs). The resulting differential photocurrent, which carries the magnetic field information, is amplified by a transimpedance amplifier (Thorlabs PDA200C), demodulated by a lock‐in amplifier (Zurich Instruments MFLI), and recorded by a data acquisition system. This differential detection architecture is pivotal for high‐sensitivity magnetometry, as it provides a high common‐mode rejection ratio to effectively suppress technical noise, such as laser intensity fluctuations. Notably, the inherent splitting asymmetry in the M‐WPS design can be readily compensated by fine‐tuning the orientation of the second QWP to equalize the optical power, thereby ensuring optimal common‐mode noise suppression.

To evaluate the dynamic range of the EPAM, we first characterized its steady‐state output response. By slowly sweeping the bias magnetic field along the *x*‐axis from −100 to +100 nT, the magnetometer's output was recorded, as shown in Figure 7b. The results reveal that the EPAM response exhibits a characteristic dispersive Lorentzian lineshape for all configurations. Specifically, the M‐WPS and C‐WPS architectures yield linear response regimes of ±2.08 and ±1.83 nT, with corresponding scale factors of 0.31 and 0.24 V/nT, respectively. This minor disparity in scale factors is primarily attributed to the different attenuation coefficients of the two designs. To further evaluate its bandwidth performance, the frequency response of the magnetometer was characterized under a 100 pTrms calibrated magnetic field (detailed in Note S10). As presented in Figure S6, the amplitude‐frequency curve yields a 3 dB bandwidth of 67 Hz. Overall, the demonstrated bandwidth range and linear response are well‐suited for biomagnetic imaging applications, such as magnetocardiography [8] and magnetoencephalography [49], where target signals typically fall within this measurement window.

Sensitivity serves as the ultimate figure of merit, defining the magnetometer's fundamental capability to resolve faint magnetic signals. To quantify this, a calibration magnetic field with a frequency of 30.5 Hz and an amplitude of 100 pTrms was applied along the sensitive *z*‐axis via a tri‐axial coil system. The output signal was sampled at 1000 Hz for 30 s, and the acquired time‐series data were converted into noise spectral density (NSD) via Fast Fourier Transform with 1 Hz interval averaging. Figure 7c displays the NSD profiles for both architectures. The magnetometers integrated with the M‐WPS and C‐WPS achieved magnetic sensitivities of 13 and 19 fT/Hz^1/2^ at 30.5 Hz, respectively. This performance is on par with conventional detection schemes relying on bulky macroscopic PBSs (7∼20 fT/Hz^1/2^) [44, 50, 51]. Although magnetometer sensitivity is influenced by various factors, benchmarking via the geometric attenuation factor demonstrates that the M‐WPS achieves a 56.7% improvement over state‐of‐the‐art metasurface splitters [24], which plays a non‐negligible role in boosting signal responsivity and ultimate sensitivity.

Notably, the sensitivity of the C‐WPS‐based system is slightly inferior to that of its monolithic counterpart. This is fundamentally attributed to the fact that the current EPAM operates within the photon shot‐noise‐limited regime, where the signal‐to‐noise ratio (SNR) is strictly governed by the photon flux incident on the PDs. Although the C‐WPS significantly enhances the CPER, the additional transmission losses inherent in its multi‐layer configuration inevitably attenuate the output intensity. Consequently, the performance penalty resulting from the reduced light throughput outweighs the SNR gains garnered from the enhanced polarization contrast. Despite this performance trade‐off, the exceptional extinction ratio and splitting symmetry of the C‐WPS hold immense potential for applications where crosstalk suppression is paramount. To validate its capability for broader polarimetric sensing beyond magnetometry, we demonstrated input polarization state reconstruction of the Stokes parameter *S*_3_ across varying ellipticity angles from 0° to 180° (detailed procedures provided in Note S11 and Figure S7). The measured values exhibit excellent agreement with theoretical predictions, yielding an average absolute error of 0.0572, thereby validating the precise detection of light ellipticity angles across a broad range using the C‐WPS and confirming its immense promise for quantum key distribution [52, 53], high‐contrast polarimetric imaging [54, 55], and circular dichroism spectroscopy.

## 4 Conclusions

In summary, we have reported an integrated polarimetry architecture based on a CLC‐WPS, fundamentally addressing the volume bottleneck of polarization detection in high‐sensitivity AMs. By leveraging the synergistic interplay between the selective Bragg reflection of patterned CLCs and TIR, we developed two application‐oriented variants: M‐WPS optimized for high efficiency and structural minimalism, and C‐WPS tailored for enhanced extinction ratios and splitting symmetry. Notably, compared to traditional PBS‐based schemes, the M‐WPS architecture reduces the optical path volume to approximately 3.3% of the original setup while achieving a high conversion efficiency of approximately 90% and a 56.7% enhancement in the geometric attenuation factor over metasurface splitters, yielding an exceptional magnetic sensitivity of 13 fT/Hz^1/2^. Furthermore, the CLC platform offers a unique competitive advantage for large‐scale, cost‐effective fabrication, as its straightforward manufacturing process avoids the need for expensive, low‐throughput nanolithography.

Looking ahead, directly bonding PDs onto the waveguide surface to construct a fully integrated polarimetric analysis unit will eliminate the physical gaps between splitting components and detectors, drastically elevating system integration. Moreover, through the precise tuning of liquid crystal parameters and grating periods, the CLC‐WPS exhibits broad versatility across a spectral range spanning from visible to near‐infrared wavelengths. Consequently, beyond AMs, our scheme can be extended to a wide array of applications requiring precision polarization analysis, such as atomic spin gyroscopes and other high‐performance quantum sensing platforms [56, 57]. Ultimately, this research facilitates the scalable production of portable, low‐cost, and high‐performance quantum sensors.

## 5 Methods

### 5.1 CLC‐WPS Fabrication

The fabrication protocol comprises two primary stages: the holographic patterning of the photoalignment layer and the subsequent coating and polymerization of the CLC precursor. Initially, a photoalignment solution was prepared by dissolving Brilliant Yellow (BY) in dimethylformamide (DMF). This solution was spin‐coated onto a cleaned 0.7‐mm‐thick glass substrate (refractive index *n* ≈ 1.57) to establish the alignment layer.

For the interference lithography, a continuous‐wave solid‐state laser (λ = 460 nm, output power: 1 W, Coherent Genesis CX‐460) served as the light source. To ensure high beam quality, the laser output was spatially filtered and expanded using a 25× microscope objective paired with a 15 µm pinhole, followed by collimation via a lens with a focal length of *f* = 80 cm. A PBS (JCOPTIX PBS25‐1) divided the collimated beam into two arms of equal intensity, with QWPs (JCOPTIX AWP10Q‐T2Q) inserted to generate orthogonal circular polarization states (LCP and RCP). The photoalignment layer was then exposed to the interfering beams at an intersection angle of 44° for 150 s, with a total optical intensity of 8.1 mW/cm^2^, defining the required horizontal periodicity.

Following the photo‐patterning, the CLC precursor mixture was spin‐coated onto the patterned substrate. The mixture comprised the photocurable mesogen RM257 (HCCH), high‐HTP chiral dopants R5011/S5011 (Helical Twisting Power ≈ 108 µm^−1^, purchased from HCCH), the photoinitiator Irgacure 184 (Macklin), and the solvent toluene (Macklin). Specific chemical compositions and spin‐coating parameters are detailed in Table S2. Finally, to fix the self‐assembled helical architecture, the coated samples were cured under UV irradiation (λ = 365 nm) at an intensity of 400 mWcm^−2^ for 10 min in a nitrogen‐purged atmosphere.

### 5.2 Magnetic Field Measurement Setup

The core sensing element of the EPAM is a cubic borosilicate glass vapor cell with external dimensions of 10 × 10 × 10 mm^3^ (internal volume: 9 × 9 × 9 mm^3^). The cell encapsulates a droplet of isotopically enriched ^87^Rb metal, buffered by 700 Torr of helium (He) and quenched by 450 Torr of nitrogen (N_2_). Due to the pressure‐induced broadening and shift from the buffer gases, the central wavelength of the ^87^Rb D1 transition is shifted to 795.0548 nm.

To attain the atomic density of approximately 10^14^ cm^−3^ requisite for the SERF regime, the vapor cell was housed within a boron nitride oven and heated to 150°C. Thermal actuation was provided by surface‐mounted, anti‐inductively wound twisted‐pair coils driven by a 200 kHz high‐frequency AC current to mitigate magnetic interference. The cell temperature was monitored by a non‐magnetic platinum resistance thermometer (PT1000) and stabilized via a PID closed‐loop control system, maintaining thermal fluctuations below 10 mK. The entire sensor head was centrally positioned within a magnetic shield assembly comprising four inner layers of mu‐metal and one outer layer of aluminum, yielding a shielding factor exceeding 10^5^. Within the shield, residual static magnetic fields were nulled to below 1 nT using a tri‐axial coil system (configured with Lee‐Whiting and saddle geometries) driven by a precision function generator (Keysight 3350B), thereby establishing the near‐zero field environment essential for SERF operation.

A DFB diode laser (Toptica DL pro) served as the light source, generating a Gaussian beam with a waist diameter of 1.5 mm. To balance optical absorption with signal strength, the laser frequency was actively locked and detuned by approximately 50 GHz from the pressure‐shifted D1 resonance line using a high‐precision wavemeter.

## Author Contributions

**Chuang Wang**: methodology, validation, Writing – original draft, visualization, data curation. **Zhibo Cui**: conceptualization, methodology, writing – original draft, software, data curation, validation. **Yishi Weng**: resources, supervision, writing – review and editing, visualization, investigation. **Gaopu Hou**: software, formal analysis, validation, data curation. **Xiangyang Zhou**: funding acquisition, writing – review and editing, project administration, resources, supervision. **Yuning Zhang**: writing – review and editing, project administration, resources, supervision, formal analysis, investigation. **Zhen Chai**: writing – review and editing, conceptualization, methodology, formal analysis, supervision, resources, funding acquisition. **Mao Ye**: resources, supervision, formal analysis, project administration, writing – review and editing. **Xu Xiao**: visualization, software, validation, writing – original draft. **Jinsheng Hu**: validation, software, formal analysis, data curation.

## Conflicts of Interest

The authors declare no conflicts of interest.

## Permissions

All included figures, tables, or text passages that have already been published elsewhere have obtained the permission from the copyright owner(s) for both the print and online format.

## Supporting information




**Supporting File**: advs77320‐sup‐0001‐SuppMat.docx.

## Data Availability

The data that support the findings of this study are available from the corresponding author upon reasonable request.

## References

[1] S. Pirandola , B. R. Bardhan , T. Gehring , C. Weedbrook , and S. Lloyd , “Advances in Photonic Quantum Sensing,” Nature Photonics 12, no. 12 (2018): 724–733, 10.1038/s41566-018-0301-6.

[2] C. L. Degen , F. Reinhard , and P. Cappellaro , “Quantum Sensing,” Reviews of Modern Physics 89, no. 3 (2017): 035002, 10.1103/RevModPhys.89.035002.

[3] J. C. Allred , R. N. Lyman , T. W. Kornack , and M. V. Romalis , “High‐Sensitivity Atomic Magnetometer Unaffected by Spin‐Exchange Relaxation,” Physical Review Letters 89, no. 13 (2002): 130801, 10.1103/PhysRevLett.89.130801. 12225013

[4] I. K. Kominis , T. W. Kornack , J. C. Allred , and M. V. Romalis , “A Subfemtotesla Multichannel Atomic Magnetometer,” Nature 422, no. 6932 (2003): 596–599, 10.1038/nature01484. 12686995

[5] H. B. Dang and M. V. Romalis , “Atomic Magnetometers for Materials Characterization,” Materials Today 14, no. 6 (2011): 258–262, 10.1016/S1369-7021(11)70140-7.

[6] D. R. Quiroz , R. J. Cooper , E. L. Foley , T. W. Kornack , G. J. Lee , and K. L. Sauer , “Interleaved NQR Detection Using Atomic Magnetometers,” Journal of Magnetic Resonance 343 (2022): 107288, 10.1016/j.jmr.2022.107288. 36209574

[7] M. J. Brookes , J. Leggett , M. Rea , et al., “Magnetoencephalography With Optically Pumped Magnetometers (OPM‐MEG): The Next Generation of Functional Neuroimaging,” Trends in Neurosciences 45, no. 8 (2022): 621–634, 10.1016/j.tins.2022.05.008. 35779970 PMC10465236

[8] Y. J. Kim , I. Savukov , and S. Newman , “Magnetocardiography With a 16‐Channel Fiber‐Coupled Single‐Cell Rb Optically Pumped Magnetometer,” Applied Physics Letters 114, no. 14 (2019): 143702, 10.1063/1.5094339.

[9] J. Ren , M. Ding , Y. Peng , et al., “A Comparative Study on the Detection and Localization of Interictal Epileptiform Discharges in Magnetoencephalography Using Optically Pumped Magnetometers Versus Superconducting Quantum Interference Devices,” Neuroimage 312 (2025): 121232, 10.1016/j.neuroimage.2025.121232. 40254146

[10] Y. Lu , T. Zhao , W. Zhu , et al., “Recent Progress of Atomic Magnetometers for Geomagnetic Applications,” Sensors 23, no. 11 (2023): 5318, 10.3390/s23115318. 37300044 PMC10256078

[11] S. Xu , C. W. Crawford , S. Rochester , V. Yashchuk , D. Budker , and A. Pines , “Submillimeter‐Resolution Magnetic Resonance Imaging at the Earth's Magnetic Field With an Atomic Magnetometer,” Physical Review A 78, no. 1 (2008): 013404, 10.1103/PhysRevA.78.013404.

[12] K. Wei , Z. Xu , Y. He , et al., “Dark Matter Search With a Resonantly‐Coupled Hybrid Spin System,” Reports on Progress in Physics 88, no. 5 (2025): 057801, 10.1088/1361-6633/adca52. 40199331

[13] X. Huang , W. Wang , Y. Hu , et al., “Double Spin Resonance for Traceable Ultrasensitive Atomic Spin Sensor,” Physical Review Letters 135, no. 4 (2025): 043001, 10.1103/gy6f-4cs9. 40794053

[14] M. Jiang , H. Su , A. Garcon , X. Peng , and D. Budker , “Search for Axion‐Like Dark Matter With Spin‐Based Amplifiers,” Nature Physics 17, no. 12 (2021): 1402–1407, 10.1038/s41567-021-01392-z.

[15] G. Liu , J. Tang , Y. Yin , Y. Wang , B. Zhou , and B. Han , “Single‐Beam Atomic Magnetometer Based on the Transverse Magnetic‐Modulation or DC‐Offset,” IEEE Sensors Journal 20, no. 11 (2020): 5827–5833, 10.1109/JSEN.2020.2973201.

[16] W. Xiao , X. Liu , T. Wu , X. Peng , and H. Guo , “Radio‐Frequency Magnetometry Based on Parametric Resonances,” Physical Review Letters 133, no. 9 (2024): 093201, 10.1103/PhysRevLett.133.093201. 39270199

[17] Y. Sebbag , A. Naiman , E. Talker , Y. Barash , and U. Levy , “Chip‐Scale Integration of Nanophotonic‐Atomic Magnetic Sensors,” ACS Photonics 8, no. 1 (2021): 142–146, 10.1021/acsphotonics.0c01473.

[18] X. Yang , M. Benelajla , S. Carpenter , and J. T. Choy , “Analysis of Atomic Magnetometry Using Metasurface Optics for Balanced Polarimetry,” Optics Express 31, no. 8 (2023): 13436, 10.1364/OE.486311. 37157482

[19] G. Zhang , H. Zeng , G. Tan , and Q. Lin , “An Integrated High‐Sensitivity VCSEL‐Based Spin‐Exchange Relaxation‐Free Magnetometer With Optical Rotation Detection,” IEEE Sensors Journal 22, no. 8 (2022): 7700–7708, 10.1109/JSEN.2022.3156814.

[20] S. Zhang , Y. Zhou , Z. Chai , Q. Liu , K. Yin , and B. Han , “Geometric‐Phase‐Lens Collimated Vertical‐Cavity Surface‐Emitting Laser Turned on Rb D₁ Line for Miniature Atomic Magnetometers,” IEEE Transactions on Instrumentation and Measurement 72 (2022): 1–7, 10.1109/TIM.2022.3232658.

[21] V. Shah , S. Knappe , P. D. D. Schwindt , and J. Kitching , “Subpicotesla Atomic Magnetometry With a Microfabricated Vapour Cell,” Nature Photonics 1, no. 11 (2007): 649–652, 10.1038/nphoton.2007.201.

[22] X. Luo , “Principles of Electromagnetic Waves in Metasurfaces,” Science China Physics, Mechanics & Astronomy 58, no. 9 (2015): 594201, 10.1007/s11433-015-5688-1.

[23] H.‐T. Chen , A. J. Taylor , and N. Yu , “A Review of Metasurfaces: Physics and Applications,” Reports on Progress in Physics 79, no. 7 (2016): 076401, 10.1088/0034-4885/79/7/076401. 27308726

[24] J. Hu , Z. Liang , P. Zhou , et al., “Integrated Optical Rotation Detection Scheme for Chip‐Scale Atomic Magnetometer Empowered by Silicon‐Rich SiN_x_ Metalens,” Optics Letters 49, no. 12 (2024): 3364, 10.1364/OL.527932. 38875621

[25] J. Zhang , S. Sun , H. Zhou , R. Zhu , R. Li , and J. Li , “Phase‐Gradient Metasurface Enables Atomic Spin chirality Detection for Elliptically Polarized Laser‐Pumped Atomic Magnetometer,” PhotoniX 6, no. 1 (2025): 29, 10.1186/s43074-025-00189-0.

[26] X. Yang , P. Mukherjee , M. Kim , et al., “Atomic Magnetometry Using a Metasurface Polarizing Beamsplitter in Silicon‐on‐Sapphire,” ACS Photonics 11, no. 9 (2024): 3644–3651, 10.1021/acsphotonics.4c00744.

[27] Y. Sebbag , E. Talker , A. Naiman , Y. Barash , and U. Levy , “Demonstration of an Integrated Nanophotonic Chip‐Scale Alkali Vapor Magnetometer Using Inverse Design,” Light: Science & Applications 10, no. 1 (2021): 54, 10.1038/s41377-021-00499-5. PMC7952415 33707424

[28] D. Budker and M. Romalis , “Optical Magnetometry,” Nature Physics 3, no. 4 (2007): 227–234, 10.1038/nphys566.

[29] J. Kitching , “Chip‐Scale Atomic Devices,” Applied Physics Reviews 5, no. 3 (2018): 031302, 10.1063/1.5026238.

[30] Y. Xu , Z. Chai , X. Meng , Y. Xu , J. Sun , and P. Zhou , “Silicon Nitride‐Photonics‐Enabled Optical Pumping for Optically Pumped Magnetometer,” Laser & Photonics Reviews 19, no. 10 (2025): 2402292, 10.1002/lpor.202402292.

[31] W. C. Griffith , S. Knappe , and J. Kitching , “Femtotesla Atomic Magnetometry in a Microfabricated Vapor Cell,” Optics Express 18, no. 26 (2010): 27167, 10.1364/OE.18.027167. 21196993

[32] M. K. Chen , Y. Wu , L. Feng , et al., “Principles, Functions, and Applications of Optical Meta‐Lens,” Advanced Optical Materials 9, no. 4 (2021): 2001414, 10.1002/adom.202001414.

[33] M. Pan , Y. Fu , M. Zheng , et al., “Dielectric Metalens for Miniaturized Imaging Systems: Progress and challenges,” Light: Science & Applications 11, no. 1 (2022): 195, 10.1038/s41377-022-00885-7. PMC9240015 35764608

[34] Z. Luo , Y. Ding , F. Peng , G. Wei , Y. Wang , and S.‐T. Wu , “Ultracompact and High‐Efficiency Liquid‐Crystal‐on‐Silicon Light Engines for Augmented Reality Glasses,” Opto‐Electronic Advances 7, no. 10 (2024): 240039, 10.29026/oea.2024.240039.

[35] X. Lu , X. Li , Y. Guo , et al., “Broadband High‐Efficiency Polymerized Liquid Crystal Metasurfaces With Spin‐Multiplexed Functionalities in the Visible,” Photonics Research 10, no. 6 (2022): 1380, 10.1364/PRJ.452272.

[36] R. Barboza , U. Bortolozzo , M. G. Clerc , and S. Residori , “Berry Phase of Light Under Bragg Reflection by Chiral Liquid‐Crystal Media,” Physical Review Letters 117, no. 5 (2016): 053903, 10.1103/PhysRevLett.117.053903. 27517773

[37] Z. Cui , J. Pan , X. Tao , et al., “Dichromatic Soliton‐Molecule Compounds in Mode‐Locked Fiber Lasers,” Laser & Photonics Reviews 18, no. 6 (2024): 2300891, 10.1002/lpor.202300471.

[38] Z. Luo , Y. Li , J. Semmen , Y. Rao , and S.‐T. Wu , “Achromatic Diffractive Liquid‐Crystal Optics for Virtual Reality Displays,” Light: Science & Applications 12, no. 1 (2023): 230, 10.1038/s41377-023-01254-8. PMC10504380 37714841

[39] Y. Pei , C. Wang , J. Qin , et al., “Ultracompact and Highly Sensitive Atomic Magnetometer Array via a Polarization Volume Grating‐Based Waveguide Structure,” ACS Photonics 12, no. 4 (2025): 1926–1935, 10.1021/acsphotonics.4c02361.

[40] Y. Ding , Y. Gu , Q. Yang , et al., “Breaking the In‐Coupling Efficiency Limit in Waveguide‐Based AR Displays With Polarization Volume Gratings,” Light: Science & Applications 13, no. 1 (2024): 185, 10.1038/s41377-024-01537-8. PMC11317523 39128902

[41] Y. Weng , Y. Zhang , J. Cui , et al., “Liquid‐Crystal‐Based Polarization Volume Grating Applied for Full‐Color Waveguide Displays,” Optics Letters 43, no. 23 (2018): 5773, 10.1364/OL.43.005773. 30499990

[42] Y. Ding , Q. Yang , Y. Li , et al., “Waveguide‐Based Augmented Reality Displays: Perspectives and Challenges,” eLight 3, no. 1 (2023): 24, 10.1186/s43593-023-00057-z.

[43] Z. Cui , Y. Wang , Y. Liu , et al., “Ultra‐Compact and High‐Precision Differential Detection Method Based on Liquid Crystal Polarization Grating for Miniature Atomic Magnetometer,” Nanophotonics 13, no. 23 (2024): 4255–4265, 10.1515/nanoph-2024-0309. 39678108 PMC11635970

[44] V. Shah and M. V. Romalis , “Spin‐Exchange Relaxation‐Free Magnetometry Using Elliptically Polarized Light,” Physical Review A 80, no. 1 (2009): 013416, 10.1103/PhysRevA.80.013416.

[45] Y. Ding , Y. Zhang , Y. Huang , et al., “Broadband and Wide‐View Cholesteric Liquid Crystal Polarization Selective Reflectors,” Optics Express 33, no. 15 (2025): 30967, 10.1364/OE.569217. 40733884

[46] Y. Weng , D. Xu , Y. Zhang , X. Li , and S.‐T. Wu , “Polarization Volume Grating With High Efficiency and Large Diffraction Angle,” Optics Express 24, no. 16 (2016): 17746, 10.1364/OE.24.017746. 27505743

[47] H. Tajvidi Safa , J. Semmen , K. Nilsen , Y. Ma , Z. Luo , and S.‐T. Wu , “Ultracompact, On‐Axis LCoS Illumination System With Local Dimming for Waveguide‐Based AR Displays,” Journal of the Society for Information Display 34, no. 5 (2026): 299–308, 10.1002/jsid.70070.

[48] Y. Ding , X. Huang , Y. Ma , Y. Li , and S.‐T. Wu , “High‐Efficiency RGB Achromatic Liquid Crystal diffractive Optical Elements,” Opto‐Electronic Advances 8, no. 3 (2025): 240181, 10.29026/oea.2025.240181.

[49] S. Rampp , H. Stefan , X. Wu , et al., “Magnetoencephalography for Epileptic Focus Localization in a Series of 1000 Cases,” Brain 142, no. 10 (2019): 3059–3071, 10.1093/brain/awz231. 31373622

[50] J. Tang , Y. Zhai , L. Cao , et al., “High‐Sensitivity Operation of a Single‐Beam Atomic Magnetometer for Three‐Axis Magnetic Field Measurement,” Optics Express 29, no. 10 (2021): 15641, 10.1364/OE.425851. 33985261

[51] Z. Liang , J. Hu , P. Zhou , et al., “Metasurface‐Integrated Elliptically Polarized Laser‐Pumped SERF Magnetometers,” Microsystems & Nanoengineering 10, no. 1 (2024): 101, 10.1038/s41378-024-00715-3. 39035364 PMC11258309

[52] G. Vest , M. Rau , L. Fuchs , et al., “Design and Evaluation of a Handheld Quantum Key Distribution Sender Module,” IEEE Journal of Selected Topics in Quantum Electronics 21, no. 3 (2015): 131–137, 10.1109/JSTQE.2014.2364131.

[53] P. Sibson , C. Erven , M. Godfrey , et al., “Chip‐Based Quantum Key Distribution,” Nature Communications 8, no. 1 (2017): 13984, 10.1038/ncomms13984. PMC5309763 28181489

[54] J. S. Tyo , D. L. Goldstein , D. B. Chenault , and J. A. Shaw , “Review of Passive Imaging Polarimetry for Remote Sensing Applications,” Applied Optics 45, no. 22 (2006): 5453, 10.1364/AO.45.005453. 16855644

[55] Q. Zhang , Y. Shang , S. Qiao , et al., “Broadband Rapid Polarization Manipulation and Imaging Based on Pancharatnam‐Berry Optical Elements,” Laser & Photonics Reviews 19, no. 5 (2025): 2401536, 10.1002/lpor.202401536.

[56] T. W. Kornack , R. K. Ghosh , and M. V. Romalis , “Nuclear Spin Gyroscope Based on an Atomic Comagnetometer,” Physical Review Letters 95, no. 23 (2005): 230801, 10.1103/PhysRevLett.95.230801. 16384290

[57] I. H. Deutsch and P. S. Jessen , “Quantum Control and Measurement of Atomic Spins in Polarization Spectroscopy,” Optics Communications 283, no. 5 (2010): 681–694, 10.1016/j.optcom.2009.10.059.

