# Supplementary material for: Efficient Cholesteric Liquid Crystal Waveguide Polarizing Splitters for High‐Sensitivity Chip‐Scale Atomic Magnetometry
Source: Adv Sci (Weinh). 2026 Aug 27:e77320. Online ahead of print. doi: 10.1002/advs.77320 (PMC13521177; doi:10.1002/advs.77320)
Supplement: Supplementary file 1 [file ADVS-9999-e77320-s001.docx]

# Supplementary Information for

Efficient Cholesteric Liquid Crystal Waveguide Polarizing Splitters for High-Sensitivity Chip-Scale Atomic Magnetometry

**Zhibo Cui^1,2,3,4#^, Chuang Wang^5#^, Xiangyang Zhou ^1,2,3,4^, Gaopu Hou^1,2,3,4^, Jinsheng Hu ^1,2,3,4^, Mao Ye^1,2,3,4,5^, Xu Xiao^1,2,3,4^, Zhen Chai^1,2,3,4,5*^, Yishi Weng^5*^ and Yuning Zhang^5^**

*^1^ Key Laboratory of Ultra-Weak Magnetic Field Measurement Technology, Ministry of Education, School of Instrumentation and Optoelectronic Engineering, Beihang University, Beijing* *100191, China*

*^2^* *Institute of Large-scale Scientific Facility and Centre for Zero Magnetic Field Science, Beihang University, Beijing 100191, China*

*^3^ Beihang Hangzhou Innovation Institute, Hangzhou 310052, China*

*^4^ Hefei National Laboratory, Hefei 230088, China*

*^5^ Joint International Research Laboratory of Information Display and Visualization, School of Electronic Science and Engineering, Southeast University, Nanjing 211189, China*

**Corresponding author: Zhen Chai, and	E-mails: zhenchai@buaa.edu.cn and*

*Yishi Weng	yishi@seu.edu.cn*

**This file includes:**

**Note S1** Comparative Analysis of Optical Layouts and System Volumetric Footprints (Figure S1)

**Note S2** Theoretical Framework of Atomic Spin Dynamics in the SERF Regime

**Note S3** Analytical Derivation of the Magnetometer Output Response (Figure S2)

**Note S4** Dielectric Tensor Formalism for Slanted Cholesteric Liquid Crystal Microstructures

**Note S5** Characterization of Angular and Spectral Response Under Standard and Optimized Polarization States (Figure S3)

**Note S6** Optical Setup for Polarization Interference Lithography (Figure S4)

**Note S7** Batch-to-Batch Device Reproducibility and Statistical Uncertainty Characterization

**Note S8** Detailed Optical Loss Budget and Photonic Trade-off Analysis

**Note S9** Experimental Wavelength Tolerance and Thermal Stability Analysis (Figure S5)

**Note S10** Magnetometer Bandwidth and Frequency Response Characterization (Figure S6)

**Note S11** Polarimetric Sensing and Input Polarization Reconstruction via Normalized Stokes Parameter (Figure S7)

**Table S1** Performance Benchmark of Beam-Splitting Architectures for Atomic Magnetometers

**Table S2** Materials and Processing Parameters for CLC-WPS Fabrication

**Table S3** Measured channel CPER values across *N* = 5 independent fabricated samples

**Table S4** Quantified optical loss budget for M-WPS and C-WPS architectures

Page: S1-S32

### Note S1 Comparative Analysis of Optical Layouts and System Volumetric Footprints

**Figure S1** Schematic comparison of detection optical layouts for various beam-splitting architectures. (a) Traditional polarizing beam splitter (PBS)-based layout, utilizing a reflective right-angle prism to achieve parallel beam output. PBS: polarizing beam splitter; PD: photodetector. (b) Metasurface-based diffractive device layout, such as a polarization-splitting metalens. (c) Free-space diffractive beam-splitting device layout, highlighting the extended propagation distance required for spatial resolution. (d) Traditional waveguide-based polarization splitter layout, illustrating the intrinsic size mismatch and the need for additional coupling optics. (e) Proposed CLC-WPS-based architecture, demonstrating minimized footprint through integrated parallel emission

The miniaturization of atomic magnetometers (AMs) is fundamentally constrained by the physical footprint occupied by the polarization detection system. To quantitatively assess the volumetric characteristics of diverse polarimetry strategies, we define two key geometric parameters assuming a fixed probe beam waist diameter of $d_{\text{beam}}=1mm$:

**Longitudinal Optical Thickness (**$h_{\text{OPT}}$**):** Defined as the total propagation length along the optical major axis extending from the front entrance surface of the polarization splitter to the effective active detection plane of the photodetectors (PDs).

**Optical Path Volume (**$V$**):** Defined as the volume of the minimum enclosing rectangular bounding box (cuboid) encompassing the entire detection light path from the beam splitter entrance to the photodetectors.

The corresponding detection optical layouts for different types of beam splitters are illustrated in Figure S1. Based on these definitions, the volumetric and thickness characteristics of five representative architectures are analyzed as follows:

**(I) Traditional PBS-based Architecture (Type A):**

Standard miniature systems typically employ a cubic polarizing beam splitter (PBS) with side length $h_{\text{PBS}}$ paired with a reflective right-angle prism of equal size $h_{\text{PBS}}$. The primary rationale for adopting this parallel layout is to route the split orthogonal beams parallel to one another, thereby facilitating subsequent light collection and differential signal processing using a balanced photodetector array and downstream differential circuitry. In this parallel layout, the total optical path volume is given by:

$$V_{\text{A}}=2h_{\text{PBS}}^{3}$$

**(II) Free-space Diffractive Polarization Splitters (Type B):**

Free-space diffractive devices (e.g., metasurfaces or liquid crystal polarization gratings) separate orthogonal polarization states via angular deflection at a first-order diffraction angle $\beta_{dif}$. In this architecture, the longitudinal optical thickness is defined as the minimum propagation distance $L_{\text{prop}}$ required to spatially resolve two 1 mm beam spots (i.e., when the center-to-center separation distance between the two split spots equals the beam diameter, $S=d_{\text{beam}}=1mm$). The volume calculated as:

$$\begin{matrix} V_{B}=d_{\text{beam}}\cdot d_{\text{beam}}\cdot L_{\text{prop}} \\ =d_{_{\text{beam}}}^{3}/\tan\left(\beta_{dif}\right) \end{matrix}$$

Although these thin-film diffractive optical elements are physically thin, the necessity for extended free-space propagation ($L_{\text{prop}}$) to resolve the split beams imposes a severe penalty on both longitudinal optical thickness and overall volumetric efficiency.

**(III) Polarization-splitting Lenses (Type C):**

This scheme incorporates focusing functionality directly into the diffractive element, where the inter-focus distance $d_{\text{foc}}$ is typically smaller than the incident beam diameter $d_{\text{beam}}$. Consequently, the longitudinal optical thickness and spatial footprint are dominated by the lens's focal length $f_{\text{lens}}$, resulting in an optical path volume of:

$$V_{\text{C}}=d_{\text{beam}}^{2}f_{\text{lens}}$$

**(IV) Traditional Integrated Waveguides (Type D):**

Conventional on-chip waveguide splitters strive for extreme micrometric miniaturization. However, their sub-micron to micrometer-scale mode profiles lead to a severe intrinsic size mismatch with the millimeter-scale vapor cells (1~10 mm) required for high-sensitivity AMs. Bypassing this mismatch necessitates additional bulk coupling optics (e.g., objective lenses, tapered fibers, and fiber arrays), which drastically inflates the effective spatial footprint and longitudinal thickness of the integrated system.

**(V) Proposed CLC-WPS Architecture (Type E):**

The proposed cholesteric liquid crystal waveguide polarization splitter (CLC-WPS) harmonizes the benefits of planar waveguide integration with the millimeter-scale spatial requirements of alkali-metal vapor cells. By achieving polarization decoupling and parallel emission within an ultrathin optical glass substrate, this architecture eliminates the need for extended free-space propagation or bulky prisms. The optical path volume for this scheme is defined as:

$$V_{\text{E}}=\left(S+d_{\text{beam}}\right)d_{\text{beam}}h_{\text{wg}}$$

where $S$denotes the beam separation distance, $h_{\text{wg}}$ represents the waveguide thickness. This design results in a radical reduction of the optical system volume compared to the aforementioned strategies.

### Table S1 Performance Benchmark of Beam-Splitting Architectures for Atomic Magnetometers

| Ref | Type | Optical thickness | OPV | Performance | | | | Fabrication | | Wave  length |
| --- | --- | --- | --- | --- | --- | --- | --- | --- | --- | --- |
|  |  |  |  | Efficiency | ER | GAF | Sensitivity^*2^ | Material | Cost^*3^ |  |
| PBS^*1^ | A | 3.2 mm | 65.5  mm^3^ | 98% | 3000 | 0.98 | 7~20 fT/Hz¹/² | Calcite | moderate | ~795 nm |
| ^[1]^ | B | 53.5 mm | 53.5  mm^3^ | 99% | 3656 | 0.99 | 14 fT/Hz¹/² | NLC/ Glass | low | ~795 nm |
| ^[2,3]^ |  | 15.3 mm | 15.3 mm^3^ | 35% | 58/14 | 0.32 | / | SOS | high | ~795 nm |
| ^[4]^ |  | 14.1 mm | 14.1  mm^3^ | 68.5% | / | <0.68 | 2.67 pT/Hz¹/² | α-Si/ SiO_2_ |  | ~795 nm |
| ^[5]^ | C | 1.9 mm | 1.9  mm^3^ | 9% | / | <0.09 | / | SiN/ SiO_2_ |  | ~795 nm |
| ^[6]^ |  | 47.7 mm | 47.7 mm3 | 30% | 5 | 0.20 | 30 fT/Hz¹/² | SiNx/ SiO_2_ |  | ~795 nm |
| ^[7,8]^ | D | / | / | 1% | 8 | 0.008 | 700 pT/Hz¹/² | SiN/SiO_2_ |  | ~780 nm |
| This  work | E | 0.7 mm | 2.15 mm^3^ | 92%/77% | 13.5/82 | 0.77 | 13 fT/Hz¹/² | CLC/ Glass | ultra-low | ~795 nm |
|  |  | 1.4 mm | 13 mm^3^ | 67%/69% | 141/134 | 0.67 | 19 fT/Hz¹/² |  |  |  |

**Note:**

| Optical Path Volume(OPV) | Extinction Ratio (ER) | Geometric Attenuation Factor (GAF) |
| --- | --- | --- |
| silicon-on-sapphire (SOS) | Nematic Liquid Crystal (NLC) | Cholesteric Liquid Crystal (CLC) |

***1: Selection Rationale for Volume and Performance of PBS:**

Following a survey of commercial off-the-shelf (COTS) micro-PBS components, the smallest standardized side length available on the market is $h_{\text{PBS}}=3.2mm$ (e.g., Foctek PBS1401, CRYLIGHT PBS1-101). Standardized COTS micro-PBS cubes smaller than 3.2 mm do not exist off the shelf due to commercial mass-production constraints such as micro-prism polishing edge chipping, dielectric coating non-uniformity on slanted surfaces, and stress-induced birefringence from precision optical bonding. Assuming $h_{\text{PBS}}=3.2mm$ and leaving necessary clear-aperture margins for $d_{\text{beam}}=1mm$, the actual minimum optical path volume for the traditional scheme is $V_{\text{A}}=2h_{\text{PBS}}^{3}=65.5mm^{3}$, with a longitudinal optical thickness of $h_{\text{OPT}}=3.2mm$. The optical efficiency (~98%) and extinction ratio (~3000) selected for the PBS baseline are typical representative values specified by commercial PBS manufacturers for performance comparison, while the reported sensitivity values are collected from representative literature.

***2: Notes on System Sensitivity:**

The cited magnetic sensitivities are collected from reported literature. Because different studies utilize distinct experimental configurations (such as vapor cell dimensions, pump light powers, electronic circuitry, and magnetic shielding layers), these values do not serve as a direct comparative metric for the intrinsic performance of the beam-splitting devices themselves. Instead, the intrinsic beam-splitting performance across different splitters can be directly benchmarked using the Geometric Attenuation Factor (GAF), which depends strictly on the device's own transmittances and extinction ratios, thereby providing an objective figure of merit independent of external system parameters.

***3: Mass Production Cost Comparison:**

Cost ratings are categorized based on material bill-of-materials (BOM) and manufacturing process overhead:

*PBS (Moderate, ~$35–60 USD/unit):* Requires multi-step optical machining (precision dicing, double-sided polishing, multi-layer dielectric coating, and dual-prism bonding) alongside an extra reflecting prism.

*LCPG (Low):* Relies on Pancharatnam–Berry geometric phase requiring strict nanometer-scale thickness control, narrowing the processing window.

*Metasurfaces & Silicon Waveguides (High):* Face critical manufacturing bottlenecks by relying on low-throughput micro/nanolithography techniques (e.g., electron-beam lithography, stepper tool time). Point-by-point patterning over large areas consumes substantial machine time, driving unit production costs extremely high.

*CLC-WPS (Ultra-Low, < $1–1.5 USD/chip):* Fabricated via roll-to-roll/spin wet coating and UV curing, fully compatible with industrial LCD production lines. Furthermore, CLC operates as a self-assembled Planar Volume Bragg Grating (PVG) maintaining high efficiency as long as film thickness exceeds 5~10 helical pitches, offering superior processing tolerance and minimal overhead.

### Note S2 Theoretical Framework of Atomic Spin Dynamics in the SERF Regime

As premier quantum sensors, AMs leverage alkali electron spin dynamics to detect infinitesimal magnetic fields. By operating in high-density vapors (10^13^~10^15^ cm⁻³) within the spin-exchange relaxation-free (SERF) regime, the spin-exchange rate significantly outpaces the Larmor precession frequency, effectively inhibiting relaxation caused by spin-exchange collisions^[9,10]^. This mechanism dramatically extends spin coherence time, enabling magnetic sensitivities to reach the sub-fT/Hz^1/2^ level, which rivals or even exceeds the performance of superconducting quantum interference devices (SQUIDs)^[11]^.

For AMs operating in the SERF regime, the complex density matrix equation can be simplified to a phenomenological Bloch equation under an external magnetic field $B$, expressed as follows^[12]^:

$$\frac{\partial P}{\partial t}=\frac{1}{q(P)}\left(\gamma_{\text{e}}P\times B+R_{\text{op}}\left(s\hat{z}-P\right)-R_{\text{rel}}P\right)$$

where$P$ represents the electron polarization vector of the Rb atoms and $q(P)$ is the nuclear slowing-down factor, which depends on the magnitude of the spin polarization. The gyromagnetic ratio of the bare electron is $\gamma_{\text{e}}\approx2\pi\times28\text{Hz}/\text{nT}$. The parameters $R_{\text{op}}$ and $R_{\text{rel}}$ represent the optical pumping rate and the total intrinsic transverse spin relaxation rate of the electron spin polarization, respectively. Typically, the photon spin component along the pumping direction can be expressed as $s=\bar{s}\cdot\hat{z}$, where $\bar{s}$ denotes the polarization state, ranging from -1 to 1. Specifically, $\bar{s}=-1$ corresponds to LCP light, $\bar{s}=0$ corresponds to linearly polarized light, and $\bar{s}=+1$ corresponds to RCP light. Thus, in the absence of polarization changes induced by light propagation through the atomic vapor cell, the average photon spin along the pumping direction of the incident light is given by^[13]^:

$$\mathbf{s}=i\left(\hat{e}\times\hat{e}^{*}\right)\cdot\hat{z}=-\sin2\varepsilon\hat{z}$$

In this work, we assume that an unknown external static magnetic field $B_{x}$ along the *x*-axis is the target to be measured. To mitigate the impact of low-frequency noise, such as laser beam vibrations, we apply an additional modulated magnetic field $B_{x}^{tot}=B_{x}+B_{mod}\cos\omega t$ along the *x*-axis to shift the signal of interest to a higher frequency, thereby facilitating precise extraction of $B_{x}$. The analytical solution for the first-harmonic component of $P_{z}$ derived from the aforementioned Bloch equation is expressed as:

$$P_{z}\left(t\right)=2\sin2\varepsilon\cdot R_{op}J_{0}(M)J_{1}(M)\frac{\gamma B_{x}}{(\gamma B_{x})^{2}+R^{2}}\sin\omega t$$

where $J_{i}(M)$ is the *i*-th order Bessel function of the first kind, and $M=\gamma B_{mod}/\left[q(P)\omega\right]$ is the modulation index, and$R=R_{\text{op}}+R_{rel}$ represents the total transverse relaxation rate. Subsequently, the signal is demodulated at the first harmonic using a lock-in amplifier, yielding the quasi-static response:

$$P_{z}=2\sin2\varepsilon\cdot R_{op}J_{0}(M)J_{1}(M)\frac{\gamma B_{x}}{(\gamma B_{x})^{2}+R^{2}}$$

### Note S3 Analytical Derivation of the Magnetometer Output Response and Proof of Dispersion Line-Shape Invariance

**Figure S2** Operating principle of the single-beam elliptically polarized atomic magnetometer

We employ the Jones matrix formalism to analyze the evolution of the light's polarization state as it interacts with the atomic vapor cell (Figure S2). First, we start from the general definition of an elliptically polarized light $E_{1}$ with ellipticity $\varepsilon$ and orientation angle, which can be expressed as:

where $E_{0}$ denotes the amplitude of the initial elliptically polarized light. Given that the polarized atomic ensemble exhibits distinct complex refractive indices for RCP and LCP light, we decompose the incident light into an orthogonal CP basis, resulting in the following expression:

$$E_{1}=E_{R}+E_{L}=E_{0}\left(A_{1}\hat{R}+B_{1}\hat{L}\right)$$

The basis vectors are defined as $\hat{R}=\left[1\text{，}-i\right]^{T}/\sqrt{2}$ and $\hat{L}=\left[1\text{，}i\right]^{T}/\sqrt{2}$, with coefficients and. After propagating through the Rb vapor cell of length $l$, the Jones vector of the output light, $E_{2}$, evolves according to the atomic medium's response:

where $k_{R}=2\pi\nu n_{R}/c$ and $k_{L}=2\pi\nu n_{L}/c$ are the complex wavenumbers of the two CP components, and $\sigma=cr_{e}f\text{Im}[L(\nu)]$ is the atomic absorption cross-section. The parameter$\theta=\left(k_{R}-k_{L}\right)l/2=\frac{\pi}{\lambda}(n_{R}-n_{L})l$ represents the magnetically induced optical rotation angle, and denotes the rotation matrix. It can be observed that the output light $E_{2}$ corresponds to an elliptically polarized beam with an orientation angle $\theta$ and ellipticity $\varepsilon$, rotated counterclockwise by an angle, and subsequently multiplied by a phase delay factor. Direct decomposition of $E_{2}$ into the CP basis reveals that the intensities of the two CP components are independent of the azimuthal angle $\theta$.

To extract the optical rotation signal through a balanced differential scheme, a QWP with its fast axis oriented at an angle relative to the *x*-axis is placed after the vapor cell. The light emerging from the QWP can then be expressed as follows:

where $A_{3}$ and $B_{3}$ represent the amplitudes of the two CP components, respectively. Since $\hat{R}$ and $\hat{L}$ are rotation-invariant bases, their magnitudes remain unaffected by the action of the rotation matrix. Direct decomposition of $E_{3}$ into the CP basis reveals that the intensities of the two CP components are independent of the azimuthal angle. For an ideal polarizing beam splitter with 100% transmission efficiency and infinite extinction ratio, the decoupled ideal intensities $I_{\text{R,ideal}}$ and $I_{\text{L,ideal}}$ for the RCP and LCP channels are expressed as:

$$\left\{\begin{matrix} I_{\text{R},\text{ideal}}=E_{0}^{2}e^{-\sigma nl}\left|A_{3}\right|^{2}=\frac{1}{2}E_{0}^{2}e^{-\sigma nl}\left[1-\cos2\varepsilon\sin2\theta\right] \\ I_{\text{L,ideal}}=E_{0}^{2}e^{-\sigma nl}\left|B_{3}\right|^{2}=\frac{1}{2}E_{0}^{2}e^{-\sigma nl}\left[1+\cos2\varepsilon\sin2\theta\right] \end{matrix}\right.$$

Consequently, the intensity difference between the two orthogonal components can be expressed as:

In SERF AMs, the optical rotation angle is typically a very small quantity ($\theta\ll1^{\circ}$). Thus, $\sin2\theta$ can be approximated as $2\theta$.

In practical miniaturized sensors incorporating a non-ideal liquid crystal beam splitter (such as the CLC-WPS), the optical performance is characterized by the actual transmission efficiencies $T_{\text{R}}$ and $T_{\text{L}}$ for the RCP and LCP channels, respectively. Due to finite circular polarization extinction ratios ($\text{CPER}_{\text{R}}$ and $\text{CPER}_{\text{L}}$), a fraction of optical power leaks into the orthogonal output channel. The power leakage coefficients $b_{\text{R}}$ and $b_{\text{L}}$ are defined by:

$$b_{\text{R}/\text{L}}=\frac{1}{\text{CPER}_{\text{R}/\text{L}}+1}$$

Under non-ideal conditions, the optical powers $I_{\text{R,real}}$ and $I_{\text{L,real}}$ detected by the two channels are no longer pure single circular polarization components, but rather linear superpositions of both orthogonal states:

$$\left\{\begin{matrix} I_{\text{R,real}}=T_{\text{R}}(1-b_{\text{R}})I_{\text{R},\text{ideal}}+T_{\text{L}}b_{\text{L}}I_{\text{L},\text{ideal}} \\ I_{\text{L,real}}=T_{\text{R}}b_{\text{R}}I_{\text{R},\text{ideal}}+T_{\text{L}}(1-b_{\text{L}})I_{\text{L},\text{ideal}} \end{matrix}\right.$$

where $I_{\text{R,ideal}}$ and $I_{\text{L,ideal}}$ represent the ideal circular polarization intensities defined in Equations (S13). Defining the effective channel transmission coefficients as $T_{\text{R}}^{'}=T_{\text{R}}(1-2b_{\text{R}})$ and $T_{\text{L}}^{'}=T_{\text{L}}(1-2b_{\text{L}})$, the resulting real differential output signal is simplified to:

The first term $\frac{1}{2}E_{0}^{2}e^{-\sigma nl}(T_{\text{L}}^{'}-T_{\text{R}}^{'})$ represents a static DC offset introduced by the optical asymmetry of the device. In the experiment, this static offset is completely suppressed by optical zeroing (by adjusting the initial QWP orientation angle $\delta_{1/4}$) or electronic differential balancing. Taking into account atomic absorption modifications and circular dichroism, the 1st-harmonic demodulated response contains both the optical rotation signal $\theta$ and a circular dichroism crosstalk term:

where $\eta_{0}$ is defined as the geometric attenuation factor, which evaluates the standalone optical beam-splitting quality of the liquid crystal device:

$$\begin{matrix} \eta_{0}=\frac{T_{\text{R}}^{'}+T_{\text{L}}^{'}}{2}\sqrt{1-\left(\frac{T_{\text{L}}^{'}-T_{\text{R}}^{'}}{\cos(2\varepsilon)(T_{\text{R}}^{'}+T_{\text{L}}^{'})}\right)^{2}} \\ =\frac{T_{\text{R}}(1-2b_{\text{R}})+T_{\text{L}}(1-2b_{\text{L}})}{2}\sqrt{1-\left(\frac{T_{\text{L}}(1-2b_{\text{L}})-T_{\text{R}}(1-2b_{\text{R}})}{\cos(2\varepsilon)\left[T_{\text{R}}(1-2b_{\text{R}})+T_{\text{L}}(1-2b_{\text{L}})\right]}\right)^{2}} \end{matrix}$$

Because the optical rotation angle $\theta$ (circular birefringence) and the absorption cross-section $\sigma$ (circular dichroism) share a common physical origin originating from the D1 transition of ^87^Rb, they are described by the real and imaginary parts of the complex Lorentzian lineshape function $L(\nu)$, respectively. Through this physical co-origin (Kramers-Kronig relation), the circular dichroism crosstalk term can be converted into a function of the optical rotation angle $\theta$:

$$\sigma nlP_{z}=2\theta\cdot\frac{\text{Im}[L(\nu)]}{\text{Re}[L(\nu)]}=2\theta\cdot\frac{\text{Δ}\nu}{2(\nu_{0}-\nu)}$$

Substituting Equation (S19) back into Equation (S17), the circular dichroism crosstalk error term is mathematically merged into the optical rotation term:

where $\xi$ is defined as the system effective attenuation factor, formulated as:

$$\begin{matrix} \xi=\eta_{0}+\frac{T_{\text{L}}^{'}-T_{\text{R}}^{'}}{4}\tan(2\varepsilon)\frac{\text{Im}[L(\nu)]}{\text{Re}[L(\nu)]} \\ =\eta_{0}+\frac{T_{\text{L}}(1-2b_{\text{L}})-T_{\text{R}}(1-2b_{\text{R}})}{8}\tan(2\varepsilon)\frac{\text{Δ}\nu}{\nu_{0}-\nu} \end{matrix}$$

Substituting Equation (S19) and the atomic spin polarization response $P_{z}$ (Equation S8 from Supplementary Note S2) into Equation (S20), the final analytical expression for the output response of the CLC-WPS-based EPAM is formulated as:

Therefore, the geometric attenuation factor $\eta_{0}$ (Eq. S18) purely reflects the intrinsic optical performance (transmissions $T_{\text{R}}$,$T_{\text{L}}$ and leakage/extinction ratios $b_{\text{R}}$, $b_{\text{L}}$) of the liquid crystal component. In contrast, the system effective attenuation factor $\xi$ (Eq. S21) incorporates both the geometric factor $\eta_{0}$ and the additional circular dichroism crosstalk induced by device asymmetry coupled with atomic absorption. Crucially, because optical absorption and dispersion are physically co-originated from the same atomic transition, this dichroism error term is seamlessly absorbed into the total system scale factor $\xi$. Therefore, employing a non-ideal liquid crystal polarizing beam splitter (such as the CLC-WPS) does not alter the intrinsic dispersive line-shape profile of the magnetometer output signal with respect to the measured magnetic field $B_{x}$; it merely introduces a scalar scaling factor $\xi$ to the overall signal amplitude.

### Note S4 Dielectric Tensor Formalism for Slanted Cholesteric Liquid Crystal Microstructures

Owing to their inherent anisotropy, CLCs possess complex dielectric properties. In general, the relative dielectric properties of an LC medium can be described by a second-order dielectric tensor matrix, expressed as:

$$\varepsilon_{r}=\left[\begin{matrix} \varepsilon_{rxx} & \varepsilon_{rxy} & \varepsilon_{rxz} \\ \varepsilon_{ryx} & \varepsilon_{ryy} & \varepsilon_{ryz} \\ \varepsilon_{rzx} & \varepsilon_{rzy} & \varepsilon_{rzz} \end{matrix}\right]$$

In a traditional planar model, the rotation of the LC optic axis is parallel to the *x-z* plane. In this case, the dielectric tensor $\varepsilon_{r0}$ is given by:

$$\varepsilon_{r0}=\left[\begin{matrix} \varepsilon_{ro}+\sin^{2}\alpha\left(\varepsilon_{re}-\varepsilon_{ro}\right) & 0 & \cos\alpha\sin\alpha\left(\varepsilon_{re}-\varepsilon_{ro}\right) \\ 0 & \varepsilon_{ro} & 0 \\ \cos\alpha\sin\alpha\left(\varepsilon_{re}-\varepsilon_{ro}\right) & 0 & \varepsilon_{ro}+\cos^{2}\alpha\left(\varepsilon_{re}-\varepsilon_{ro}\right) \end{matrix}\right]$$

where $\varepsilon_{ro}=n_{o}^{2}$ and $\varepsilon_{re}=n_{e}^{2}$ denote the relative dielectric constants for ordinary and extraordinary waves, respectively. The azimuthal angle $\alpha$ is defined as per Section 2.2.

In the slanted CLC model, the microscopic helical axis is tilted by an angle $\beta$ relative to the laboratory coordinate system, while the macroscopic boundaries remain unchanged. Mathematically, this physical configuration is represented by a coordinate rotation of the relative dielectric tensor matrix. The transformation matrix $Q$ is defined as:

$$Q=\left[\begin{matrix} \cos\beta& -\sin\beta& 0 \\ \sin\beta& \cos\beta& 0 \\ 0 & 0 & 1 \end{matrix}\right]$$

According to the laws of coordinate transformation, the relative dielectric tensor matrix for the slanted PVG in the new coordinate system is given by $\varepsilon_{r1}=Q^{-1}\varepsilon_{r0}Q$:

$$\begin{matrix} \varepsilon_{r1}=Q^{-1}\varepsilon_{r0}Q \\ =\left[\begin{matrix} \varepsilon_{ro}+\cos^{2}\beta\sin^{2}\alpha\left(\varepsilon_{re}-\varepsilon_{ro}\right) & -\cos\beta\sin\beta\sin^{2}\alpha\left(\varepsilon_{re}-\varepsilon_{ro}\right) & \cos\alpha\sin\alpha\cos\beta\left(\varepsilon_{re}-\varepsilon_{ro}\right) \\ -\cos\beta\sin\beta\sin^{2}\alpha\left(\varepsilon_{re}-\varepsilon_{ro}\right) & \varepsilon_{ro}+\sin^{2}\beta\sin^{2}\alpha\left(\varepsilon_{re}-\varepsilon_{ro}\right) & -\cos\alpha\sin\alpha\sin\beta\left(\varepsilon_{re}-\varepsilon_{ro}\right) \\ \cos\alpha\sin\alpha\cos\beta\left(\varepsilon_{re}-\varepsilon_{ro}\right) & -\cos\alpha\sin\alpha\sin\beta\left(\varepsilon_{re}-\varepsilon_{ro}\right) & \varepsilon_{ro}+\cos^{2}\alpha\left(\varepsilon_{re}-\varepsilon_{ro}\right) \end{matrix}\right] \end{matrix}$$

### Note S5 Comparative Angular and Spectral Response Under Standard and Optimized Polarization States

**Figure S3** Simulated angular and spectral response of the CLC-WPS under different incident polarization states. **(a, c)** Diffraction efficiency maps as a function of incident angle and wavelength under standard left-circularly polarized (LCP) illumination defined by the Jones vector parameters . A slight deviation in the peak response from the design target (795 nm at normal incidence) is observed due to the symmetry breaking of the slanted grating. **(b, d)** Corresponding diffraction efficiency maps under the optimized elliptically polarized illumination defined by . The peak efficiency is perfectly restored to the target wavelength of 795 nm at 0° incidence, confirming that matching the incident polarization to the grating's eigenmode effectively compensates for the structural asymmetry

### Note S6 Optical Setup for Polarization Interference Lithography

**Figure S4** Schematic of the dual-beam polarization interference exposure setup. A spatially filtered and collimated laser beam is split into two arms of equal intensity by a polarizing beam splitter (PBS). Quarter-wave plates (QWPs) modulate the beams into orthogonal circular polarization states (LCP and RCP). The beams intersect at the sample plane at an angle of 44°, generating a polarization interference pattern that encodes the periodic anchoring required for the CLC microstructure

### Table S2 Materials and Processing Parameters for CLC Fabrication

| Type | Solute | Solvent | Concentration | Coating Speed(rpm) | Coating time(s) |
| --- | --- | --- | --- | --- | --- |
|  | BY | DMF | 0.4% | 2000 | 30 |
| CLC R/S | R5011/S5011 (1.43 wt.%), Irgacure 651 (1.82 wt.%), and RM257 (96.74 wt.%) | Toluene | 40 wt.% | 800 | 20 |

### Note S7 Batch-to-Batch Device Reproducibility and Statistical Uncertainty Characterization

To rigorously evaluate the manufacturing reproducibility, batch-to-batch consistency, and statistical uncertainty of the proposed cholesteric liquid crystal waveguide polarization splitters (CLC-WPS), we fabricated and optically characterized a randomized batch of *N*=5 independent devices for both the monolithic (M-WPS) and cascaded (C-WPS) architectures. All test samples were prepared under standardized laboratory protocols, encompassing holographic photoalignment exposure, spin-coating of the photocurable LC precursor, thermal annealing, UV-induced photopolymerization, and precision optical adhesive lamination.The circular polarization extinction ratio (CPER) for both the left-circularly polarized (LCP) and right-circularly polarized (RCP) output channels was systematically measured using a precision optical characterization setup consisting of a rotating quarter-wave plate (QWP) paired with a linear polarizer (LP) and two calibrated optical power meters (Thorlabs PM120VA). The experimental results across the *N*=5 independent fabricated samples are summarized in Supplementary Table S3.As detailed in Supplementary Table S3, both architectures exhibit remarkably low standard deviations across independent fabrication runs, confirming exceptional device-to-device repeatability.

### Table S3 Measured channel CPER values across *N* = 5 independent fabricated samples

| Architecture | Sample ID | | RCP channel CPER | LCP channel CPER |
| --- | --- | --- | --- | --- |
| M-WPS | | Sample 1 | 14.1 | 81.8 |
|  |  | Sample 2 | 12.2 | 76.8 |
|  |  | Sample 3 | 14.8 | 87.5 |
|  |  | Sample 4 | 12.6 | 78.1 |
|  |  | Sample 5 | 13.5 | 82.0 |
| C-WPS | | Sample 1 | 142.5 | 135.8 |
|  |  | Sample 2 | 133.0 | 128.2 |
|  |  | Sample 3 | 148.2 | 141.0 |
|  |  | Sample 4 | 136.1 | 129.5 |
|  |  | Sample 5 | 141.0 | 134.0 |

### Note S8 Detailed Optical Loss Budget and Photonic Trade-off Analysis

To elucidate the throughput discrepancy between the monolithic (M-WPS, ~90% throughput) and cascaded (C-WPS, ~70% throughput) architectures, we established a quantitative optical loss budget along their respective waveguiding trajectories. In the M-WPS, guided light undergoes a single in-coupling diffraction at the bottom CLC film, one total internal reflection (TIR) bounce at the top glass-air interface, and a single vertical out-coupling diffraction. Conversely, the C-WPS incorporates dual CLC filtering layers (R-CLC and S-CLC) bonded by an intermediate ~30 μm NOA61 layer; to satisfy geometric symmetry and spatial beam separation, the guided beam executes two complete TIR cycles across the stacked sandwich structure, traversing the optical adhesive layer and adhesive-glass interfaces a total of 6 times prior to vertical out-coupling.

Each individual loss channel along these paths was quantified through targeted experimental calibrations and numerical models. The grating diffraction efficiencies were measured using a precision optical power meter (Thorlabs PM120VA) referenced against a blank glass substrate, yielding an in-coupling efficiency of ~91.5% for the single S-CLC layer in M-WPS, and ~89.2% (R-CLC) / ~88.4% (S-CLC) for C-WPS. RCWA and 3D-FEM simulations confirm that the optimized slanted Bragg profile achieves an out-coupling extraction efficiency exceeding 95%. Within the polymer films, intrinsic material haze and scattering were determined at ~0.85% per layer (EVERFINE HAM-300). For the C-WPS, the NOA61 bonding layer ($n\approx1.56$) introduces an absorption loss of ~0.13% per pass at 795 nm (~0.8% total over 6 passes), while Fresnel reflections at the NOA61-glass ($n\approx1.57$) and glass-air interfaces contribute ~0.1% per event (~0.6% total over 6 passes).

Multiplying these sequential efficiency factors along their respective optical paths yields calculated net throughputs of ~90.5% for M-WPS and ~73.1% for C-WPS, matching our experimental end-to-end measurements (~90% and ~70%) within tight margins, as summarized in Supplementary Table S4. This loss budget transparently elucidates the photonic trade-off: M-WPS minimizes optical interfaces to maximize throughput and photon-shot-noise-limited magnetic sensitivity (13 fT/Hz^1/2^), whereas C-WPS sacrifices ~20% throughput via dual-stage filtering to achieve superior circular polarization contrast (141/134 ER) and spatial beam symmetry.

## Table S4 Quantified optical loss budget for M-WPS and C-WPS architectures

| Loss mechanism / Efficiency channel | M-WPS | C-WPS |
| --- | --- | --- |
| In-coupling/Out-coupling diffraction efficiency | 91.5% | 89.2%(R-CLC)  /88.4%(S-CLC) |
| TIR loss | ~0.1%*1 bounce | ~0.1%*2 bounce |
| CLC absorption/scattering loss | ~0.85%*1 layer | ~0.85%*2 layer |
| Adhesive absorption loss | NA | ~0.8%*6 passes |
| Adhesive/glass interface loss | NA | ~0.1%*6 passes |
| Calculated Cumulative Efficiency | ~90.5% | ~73.1% |
| Measured overall throughput | ~90% | ~70% |

### Note S9 Experimental Wavelength Tolerance and Thermal Stability Analysis

**Figure S5** Measured optical diffraction efficiency spectrum of the polymerized R-CLC grating layer, demonstrating a broad full-width at half-maximum bandwidth of ~65 nm around the 795 nm operating wavelength

In single-beam elliptically polarized atomic magnetometers (EPAMs), the laser frequency is actively locked to the 795 nm ^87^Rb D1 transition line and detuned by ~50 GHz (~0.1 nm) to balance optical absorption with signal responsivity. To verify the practical spectral tolerance of the fabricated M-WPS around 795 nm, its diffraction efficiency spectrum was measured using a fiber-optic spectrometer (Fuxiang R1) coupled with a broadband tungsten-halogen optical source.

As shown in Supplementary Figure S5, the photopolymerized WPS exhibits a remarkably broad full-width at half-maximum (FWHM) response bandwidth of approximately 65 nm (spanning from 762 nm to 827 nm) centered at 795 nm. Physically, this broad reflection bandwidth is fundamentally governed by the large birefringence of the reactive mesogen ($\text{Δ}n\approx0.18$), where the photonic bandgap width scales as $\text{Δ}\lambda=p\cdot\text{Δ}n$ ($p$ being the helical pitch). Because the operational spectral bandwidth of the M-WPS ($\text{Δ}\lambda_{\text{CLC}}\approx65\text{nm}$) is nearly three orders of magnitude broader than typical diode laser frequency drifts or thermal mode-hop detunings (0.1 nm), the optical throughput and polarization-splitting efficiency remain exceptionally high and flat across the operational band, rendering the device completely immune to laser wavelength fluctuations.

### Note S10 Magnetometer Bandwidth and Frequency Response Characterization

**Figure S6** Normalized amplitude-frequency response of the atomic magnetometer measured under a 100 pTrms calibrated AC magnetic field from 1 Hz to 100Hz

The magnetometer's bandwidth is a critical parameter for characterizing its dynamic performance, especially since biomagnetic signals from magnetoencephalography (MEG) and magnetocardiography (MCG) typically reside within the 1~100 Hz frequency range. To confirm adequate frequency coverage, we measured the magnetometer's amplitude response from 1 Hz to 100 Hz under a 100 pTrms calibrated magnetic field. The resulting amplitude-frequency response data were subsequently fitted with a first-order low-pass filter model:

$$R(f)=\frac{A}{\sqrt{(f-f_{0})^{2}+B^{2}}}+C$$

where $A$ represents the response coefficient, $f_{0}$ denotes the center frequency, $B$ represents the bandwidth parameter, and $C$ denotes the baseline offset response. Supplementary Figure S7 shows the normalized amplitude-frequency response curve. From the fitting, the 3 dB bandwidth of the atomic magnetometer is determined to be 67 Hz. Because biological magnetic signals generally reside within 100 Hz, the measured 67 Hz bandwidth fully satisfies the requirements for practical biomagnetic sensing applications.

### Note S11 Input Polarization Reconstruction via Normalized Stokes Parameter

**Figure S7** Comparison between reconstructed experimental Polarization Index (PI) and theoretical Stokes parameter $S_{3}$ across the continuous ellipticity angle(0° to 180° in steps of 5°). The solid curve with open squares represents the theoretical $S_{3}=\sin(2\varepsilon)$, while the solid circles denote the experimental PI derived from dual-channel optical power measurements.

To quantitatively demonstrate the high-accuracy polarization state reconstruction capability of the C-WPS, we performed an input polarization state reconstruction using the experimental dataset corresponding to Figure 5c. A collimated 795 nm Gaussian laser beam was modulated by a linear polarizer (LP) and a quarter-wave plate (QWP) to produce incident light with a continuously tunable ellipticity angle $\varepsilon$ (0° to 180° in steps of 5°). The decoupled optical powers from the right and left output channels of the C-WPS were simultaneously recorded using two synchronized optical power meters (Thorlabs PM120VA).

In order to quantitatively describe the incident polarization state based on the output signals of the dual channels, we define a normalized Polarization Index (PI), evaluated as:

$$\text{PI}=\frac{P_{\text{RCP}}-P_{\text{LCP}}}{P_{\text{RCP}}+P_{\text{LCP}}}$$

where $P_{\text{RCP}}$ and $P_{\text{LCP}}$ refer to the optical powers extracted from the right and left channels of the C-WPS, respectively. Theoretically, PI corresponds strictly to the normalized third Stokes parameter $S_{3}=\sin(2\varepsilon)$, which quantifies the normalized optical power difference between right- and left-circularly polarized components.

The comparison between the reconstructed experimental PI values and the theoretical $S_{3}=\sin(2\varepsilon)$ curve is presented in Supplementary Figure S6. The experimental PI matches the theoretical $S_{3}$ curve with high fidelity across all non-zero operational sampling points yielding an average absolute error of 0.0572 and an average relative error of 11.67%.

The main reconstruction discrepancies are predominantly concentrated near the peak response regions (extrema near $\varepsilon$= 45° and 135°). This behavior originates from the intrinsic symmetry breaking of the slanted cholesteric liquid crystal architecture. As simulated in Section 2.2 and experimentally verified in Section 3.2 of the main text, the tilted helical matrix induces a slight shift in peak coupling efficiency away from the ideal pure circular polarization states. Crucially, by systematically measuring and compensating for this intrinsic angular offset during calibration, the reconstruction accuracy can be further refined for even more precise polarization state testing.

## References

[1] Z. Cui, Y. Wang, Y. Liu, M. Jin, J. Sun, Y. Zhai, X. Zhou, Z. Chai, *Nanophotonics* **2024**, 13, 4255.

[2] X. Yang, M. Benelajla, S. Carpenter, J. T. Choy, *Opt. Express* **2023**, 31, 13436.

[3] X. Yang, P. Mukherjee, M. Kim, H. Mei, C. Fang, S. Choi, Y. Tong, S. Perlowski, D. A. Czaplewski, A. M. Dibos, M. A. Kats, J. T. Choy, *ACS Photonics* **2024**, 11, 3644.

[4] J. Zhang, S. Sun, H. Zhou, R. Zhu, R. Li, J. Li, *PhotoniX* **2025**, 6, 29.

[5] Y. Xu, Y. Xu, J. Sun, Y. Mao, Z. Chai, J. Li, *Adv. Opt. Mater.* **2024**, 12, 2301353.

[6] J. Hu, Z. Liang, P. Zhou, L. Liu, G. Hu, P. Du, M. Ye, *Opt. Lett.* **2024**, 49, 3364.

[7] Y. Sebbag, A. Naiman, E. Talker, Y. Barash, U. Levy, *ACS Photonics* **2021**, 8, 142.

[8] Y. Sebbag, E. Talker, A. Naiman, Y. Barash, U. Levy, *Light Sci. Appl.* **2021**, 10, 54.

[9] J. C. Allred, R. N. Lyman, T. W. Kornack, M. V. Romalis, *Phys. Rev. Lett.* **2002**, 89, 130801.

[10] M. P. Ledbetter, I. M. Savukov, V. M. Acosta, D. Budker, M. V. Romalis, *Phys. Rev. A* **2008**, 77, 033408.

[11] I. K. Kominis, T. W. Kornack, J. C. Allred, M. V. Romalis, *Nature* **2003**, 422, 596.

[12] H. B. Dang, A. C. Maloof, M. V. Romalis, *Appl. Phys. Lett.* **2010**, 97, 151110.

[13] G. Vasilakis, *Precision Measurements of Spin Interactions with High Density Atomic Vapors*, Ph.D. thesis, Princeton University, **2011**.
